# Subcortical Source and Modulation of the Narrowband Gamma Oscillation in Mouse Visual Cortex

**DOI:** 10.1016/j.neuron.2016.12.028

**Published:** 2017-01-18

**Authors:** Aman B. Saleem, Anthony D. Lien, Michael Krumin, Bilal Haider, Miroslav Román Rosón, Asli Ayaz, Kimberly Reinhold, Laura Busse, Matteo Carandini, Kenneth D. Harris

**Affiliations:** 1 UCL Institute of Ophthalmology, University College London, London WC1E 6BT, UK; 2 UCL Institute of Neurology, University College London, London WC1E 6BT, UK; 3 Department of Neuroscience, Physiology, and Pharmacology, University College London, London WC1E 6BT, UK; 4 Division of Biological Sciences, University of San Diego, San Diego, CA 92110, USA; 5 Werner Reichardt Centre for Integrative Neuroscience, University of Tübingen, 72074 Tübingen, Germany; 6 Graduate Training Centre of Neuroscience, University of Tübingen, 72074 Tübingen, Germany

**Keywords:** gamma, primary visual cortex, mouse vision, thalamus, neural circuits, lateral geniculate nucleus

## Abstract

Primary visual cortex exhibits two types of gamma rhythm: broadband activity in the 30–90 Hz range and a narrowband oscillation seen in mice at frequencies close to 60 Hz. We investigated the sources of the narrowband gamma oscillation, the factors modulating its strength, and its relationship to broadband gamma activity. Narrowband and broadband gamma power were uncorrelated. Increasing visual contrast had opposite effects on the two rhythms: it increased broadband activity, but suppressed the narrowband oscillation. The narrowband oscillation was strongest in layer 4 and was mediated primarily by excitatory currents entrained by the synchronous, rhythmic firing of neurons in the lateral geniculate nucleus (LGN). The power and peak frequency of the narrowband gamma oscillation increased with light intensity. Silencing the cortex optogenetically did not abolish the narrowband oscillation in either LGN firing or cortical excitatory currents, suggesting that this oscillation reflects unidirectional flow of signals from thalamus to cortex.

## Highlights

• Mouse V1 exhibits a pronounced narrowband gamma oscillation close to 60 Hz
• This oscillation is strongest in layer 4 and specific to excitatory currents
• It increases with arousal and light intensity and decreases with visual contrast
• It is seen in lateral geniculate neurons, regardless of V1 activity

## Teaser

Saleem et al. discover that the narrowband gamma oscillation close to 60 Hz prevalent in the mouse visual cortex is inherited from the visual thalamus. The oscillation is enhanced by arousal and light intensity, and suppressed by visual contrast.

## Introduction

Gamma rhythms are produced by a wide range of brain circuits and are thought to reflect multiple phenomena. Rhythms in a broad gamma range (30–90 Hz) have long been observed in regions including isocortex (Engel et al., 2001, Fries, 2009), hippocampus (Bragin et al., 1995), amygdala, striatum (Popescu et al., 2009), and cerebellum (Middleton et al., 2008). In cortex, these rhythms are believed to arise from the precisely timed synchronization of inhibitory networks (Cardin et al., 2009, Penttonen et al., 1998, Sohal et al., 2009, Wang and Buzsáki, 1996) and have been implicated in a wide range of functions including coherent transmission of information between neuronal assemblies (Fries, 2005, Fries, 2009), multiplexing of information (Lisman and Idiart, 1995), or binding of multiple features of a sensory scene (Singer, 1999). Recently, however, it has become clear that gamma rhythms reflect multiple phenomena, and that a single neuronal circuit can support multiple types of gamma rhythms. In hippocampal area CA1, for example, gamma rhythms occurring in distinct frequency bands are coherent with distinct input sources (Colgin et al., 2009), perhaps to provide separate routes for information from those structures. Understanding the origin of multiple patterns of high-frequency rhythms is essential for understanding the functional roles these rhythms might play.

In the visual cortex, there is evidence for two types of gamma rhythm, one with power distributed in a band between 30 and 90 Hz, and one that oscillates in a much narrower band. The gamma rhythms between 30 and 90 Hz have long been described in multiple species including cats (Eckhorn et al., 1988, Gray and Singer, 1989), primates (Fries et al., 2001, Kreiter and Singer, 1992, Pesaran et al., 2002), humans (Tallon et al., 1995), and mice (Cardin et al., 2009, Sohal et al., 2009, Welle and Contreras, 2016). We refer to these rhythms between 30 and 90 Hz as broadband gamma. They are modulated by factors such as stimulus position, context, or cognitive state (Chalk et al., 2010, Fries et al., 2001, Gieselmann and Thiele, 2008, Gray and Singer, 1989, Pesaran et al., 2002). A fundamentally different gamma oscillation has been reported in visual cortex of anesthetized cats. This oscillation is extremely narrow in bandwidth in single experiments, and can appear also in the lateral geniculate nucleus (LGN) and retina (Koepsell et al., 2009, Koepsell et al., 2010, Neuenschwander and Singer, 1996). A gamma oscillation with a sharp band close to 60 Hz has also been observed in visual cortex of awake mice, where its power grows markedly with locomotion (Lee et al., 2014, Niell and Stryker, 2010). We refer to this oscillation as narrowband gamma, but note that some authors have used this term differently (Hermes et al., 2015a, Hermes et al., 2015b, Ray and Maunsell, 2010, Ray and Maunsell, 2011, Scheeringa et al., 2011, Winawer et al., 2013). The origins of this oscillation, and the behavioral and sensory factors modulating it, are unknown.

Here we investigate the narrowband gamma oscillation in awake mouse primary visual cortex (V1) and establish the factors that determine its strength and frequency, and its relationship to broadband gamma activity. We find that this oscillation has different properties from broadband gamma activity: its amplitude increases with mean light intensity and with locomotion, but it decreases with visual contrast. The narrowband oscillation occurs independently of broadband gamma activity, indicating that it arises from different mechanisms. These mechanisms involve synaptic excitation more than inhibition and operate before the visual signals reach the cortex. Indeed, the narrowband oscillation is present earlier in the visual system, arising at least as early as the LGN.

## Results

We start by characterizing the narrowband gamma oscillation in mouse V1. We then explore its synaptic basis and its dependence on visual contrast and locomotion. Finally, we turn to the LGN and to optogenetic manipulations that investigate the source of the narrowband oscillation.

### Narrowband Gamma in V1

**Figure 1:**
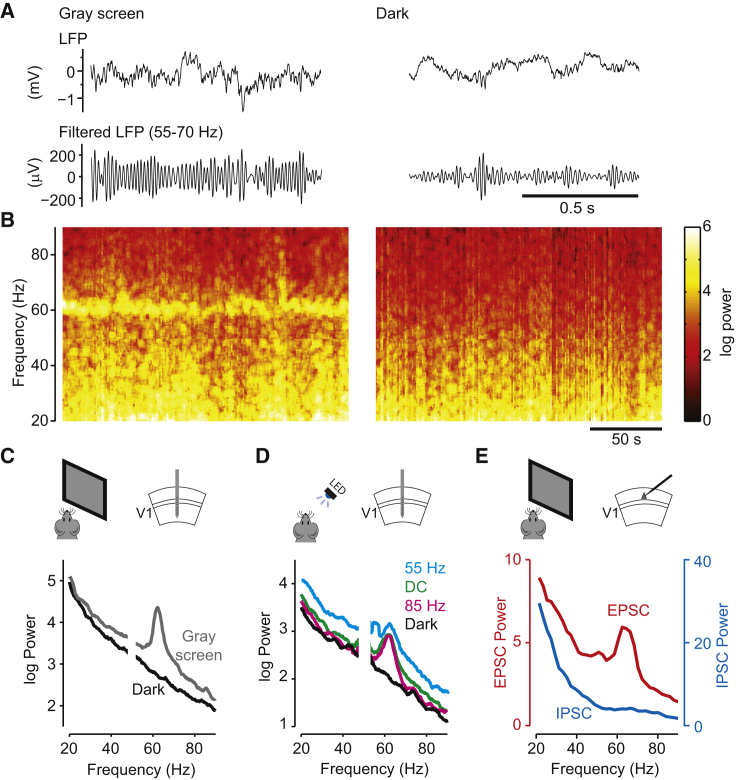
Narrowband Gamma in Mouse Visual Cortex (A) Examples of local field potential (LFP) recorded in the primary visual cortex (V1) of mice running on a treadmill while viewing a uniform gray screen (left) or in complete darkness (right). Top traces: 1 s of single-trial broadband LFP; lower traces: same data, filtered between 55 and 70 Hz. (B) Spectrograms showing the variation of LFP power over a longer timescale. The LFP was recorded in V1 of mice running on a treadmill during uniform gray (left) or complete darkness (right). (C) Average LFP power spectra during uniform gray screen conditions (gray) or complete darkness (black) (same recording shown in B). (D) LFP power spectra under various conditions of LED stimulation: flickering at 55 Hz (cyan), flickering at 85 Hz (magenta), constantly on (DC, green), and off (Dark, black) (example from one recording session). (E) Power in the excitatory postsynaptic currents (EPSCs, red; whole-cell voltage-clamp recording at −70 mV) and inhibitory postsynaptic currents (IPSCs, blue; whole-cell voltage-clamp recording at +20 mV) in layer 2/3 neurons in V1, recorded during the uniform gray condition (average spectra across eleven recorded neurons).

Consistent with previous reports (Lee et al., 2014, Niell and Stryker, 2010), when we recorded in V1 of awake mice using multi-electrode arrays (Ayaz et al., 2013, Saleem et al., 2013) we observed a sharp peak in the local field potential (LFP) power spectrum close to 60 Hz (Figures 1A–1C and S1, available online). The frequency of this oscillation was 61 Hz in this example experiment (Figures 1B and 1C) and could vary between 55 and 70 Hz in other experiments, with a remarkably narrow bandwidth of 2–5 Hz. This oscillation could not have reflected mains interference, for these recordings were conducted in Europe, where (unlike in the United States) the electricity alternates at 50 Hz.

This narrowband oscillation was prominent when the mice viewed a uniform gray screen (50 cd/m^2^), but it disappeared when we repeated the experiments in complete darkness (Figures 1A–1C; <10^−2^ cd/m^2^). The oscillation was due to the overall light intensity, not the monitor refresh frequency (which can weakly entrain visual cortical neurons; Veit et al., 2011, Williams et al., 2004). We confirmed this with control experiments where we stimulated the visual field not with a monitor, but with a light-emitting diode (LED, 470 nm) generating light steadily with direct current (DC) input, or flickering at different rates. We saw a narrowband oscillation close to 60 Hz in all cases, independent of visual flicker rates (Figure 1D; p > 0.1, t test for all pairs of flicker rates; see Supplemental Experimental Procedures).

Unlike broadband gamma rhythms, which involve inhibitory currents (Cardin et al., 2009, Hasenstaub et al., 2005, Sohal et al., 2009, Welle and Contreras, 2016), the narrowband gamma oscillation was primarily mediated by excitatory synaptic currents (EPSCs) (Figure 1E). To uncover the synaptic basis of the narrowband oscillation, we analyzed intracellular recordings from layer 2/3 regular-spiking (putative pyramidal) neurons in V1 of awake mice viewing a uniform gray screen (Haider et al., 2013) (Figure 1E). EPSCs, isolated by voltage clamping the membrane potential near the reversal potential for inhibition, showed a clear peak around 60 Hz, similar to that seen in the extracellular LFP. The power in the narrowband gamma frequency range was significantly greater than a baseline prediction based on a smooth power spectrum that excludes this range (the “residual spectrum”; Figure S1; p = 0.027; t test over n = 11 recordings). However, we did not observe a similar peak in narrowband gamma power in the inhibitory synaptic currents (IPSCs) recorded from the same neurons during the same stimulus conditions (p = 0.11; n = 11 recordings). This suggests that at least in superficial layers, the narrowband gamma oscillation is primarily mediated by EPSCs.

### Narrowband Gamma Is Suppressed by Visual Contrast

**Figure 2:**
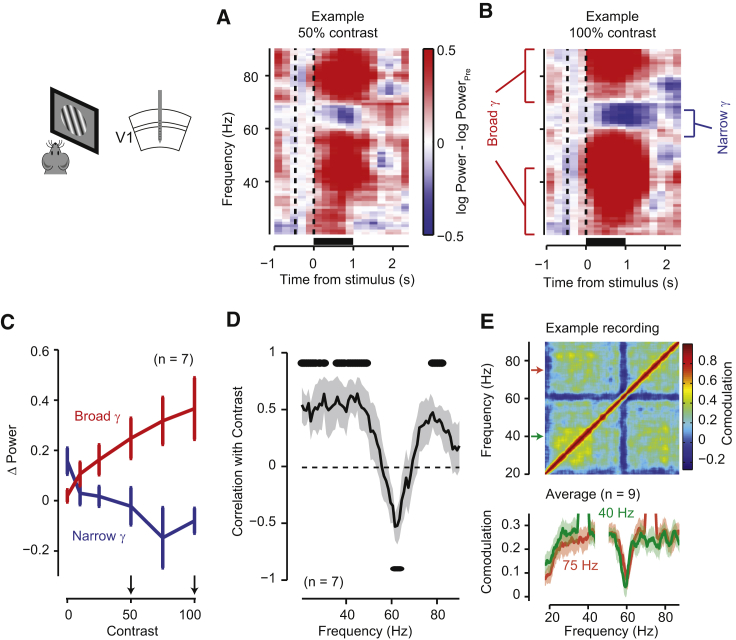
Narrowband Gamma Oscillations Are Suppressed by Visual Contrast (A and B) LFP spectrogram averaged over 20 presentations of a drifting grating stimulus at 50% contrast (A) and 100% contrast (B) in a single experiment. Color shows power relative to pre-stimulus baseline (−0.5 to 0 s); bars below indicate the period of presentation of a 50° sinusoidal drifting grating in the retinotopic location of the recorded area. (C) Power at the end of the stimulus presentation period (1 s) as a function of visual contrast for narrowband gamma (blue, calculated as the mean across the 55–65 Hz band) and broadband gamma (red, calculated as the mean across bands 20–40 and 70–90 Hz). The small increase in narrowband gamma power for zero contrast (blank) stimuli likely reflects continuing recovery from suppression by the preceding stimulus. Error bars show mean ± SEM. (D) Correlation of power at different frequencies with contrast. Shaded area shows the mean ± SEM across experiments (n = 7); dots above/below the curve indicate frequencies with significant positive/negative correlations (p < 0.05; n = 7). (E) Comodulation of power at different frequencies, i.e., the correlation in the power of different frequency bands, in an example recording. The blue cross visible at 60 Hz indicates that narrowband gamma power is uncorrelated with broadband gamma power. Bottom: comodulation of two frequencies of broadband gamma, 40 Hz (green) and 75 Hz (red), with respect to other frequency bands, showing a dip at narrowband gamma frequency. Shaded area shows the mean ± SEM across nine recordings in a virtual reality environment. Arrows indicate the frequencies shown in the bottom plots.

Another fundamental difference between narrowband and broadband gamma activity is that increasing visual contrast had opposite effects on them (Figures 2A–2D and S2). When we measured V1 responses in awake mice to drifting gratings (60° diameter, 2 cycles/s, 0.05 cycles/°) at different levels of visual contrast, we found a positive correlation of broadband power with contrast (ρ = 0.59 ± 0.18; n = 7 recordings; Figures 2A–2D). For the narrowband gamma oscillation, instead, the power was instead negatively correlated with contrast (ρ = −0.43 ± 0.16; n = 7 recordings; Figures 2C and 2D).

Narrowband gamma oscillation was suppressed by contrast not only during passive visual presentation, but also while mice used visual inputs to guide locomotion (Figure 2C). We recorded V1 activity while mice navigated in a virtual reality environment where visual cues indicated a position that mice must reach to receive a water reward. The narrowband gamma oscillation increased prominently during inter-trial periods, when the screen was uniform gray (Figure 2C; the increase in narrowband power prior to the offset of the previous trial likely reflects the uniform gray texture of the wall at the far end of the virtual corridor). Further, throughout the experiment the power of this narrowband gamma oscillation was uncorrelated with power in broadband gamma range (Figure 2E).

### Narrowband Gamma in the LGN

**Figure 3:**
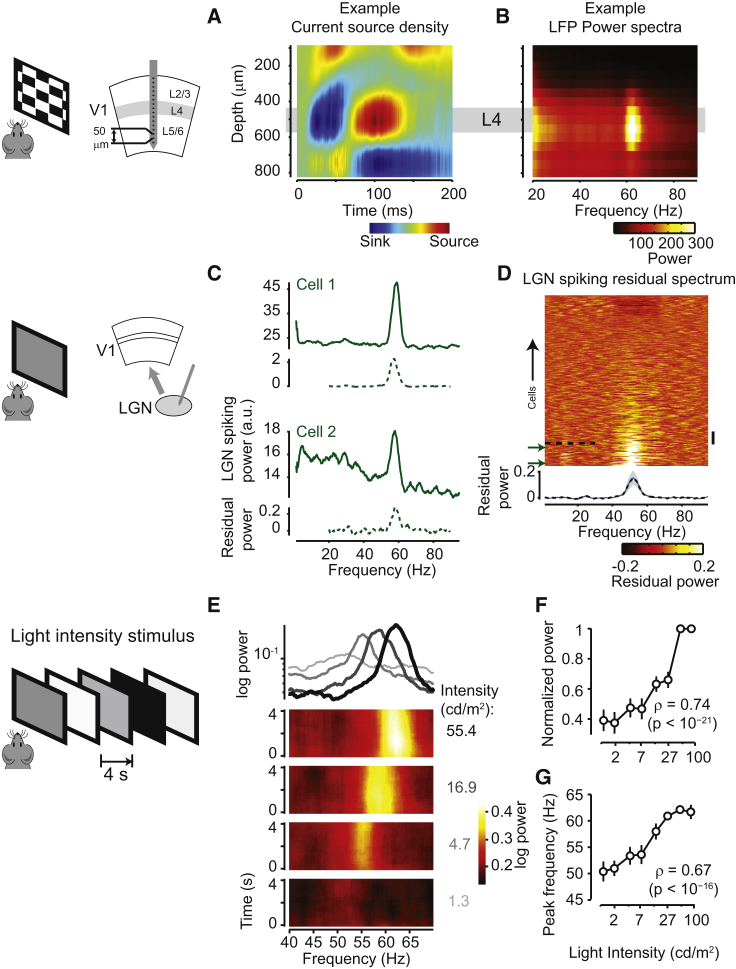
Narrowband Gamma Is Highest in L4 of V1 and Present in LGN Spiking Activity (A) We recorded across the laminae of V1 using a 16-channel multi-electrode array. Current source density analysis was used to identify L4 as the location of the earliest current sink in response to a checkerboard stimulus (figure shows data from one representative experiment). (B) LFP power spectrum as a function of depth in this same experiment, showing highest narrowband gamma power close to L4. (C) Extracellular recordings from the LGN using a multi-electrode array. The spike train power spectra show a clear gamma modulation (two example neurons). Below each spectrum, the dotted line shows the fractional increase in power from a fit to the spectra excluding the narrowband gamma range (residual power spectrum). (D) Pseudocolor plot showing residual spectra of all recorded LGN neurons (n = 323), ordered by narrowband gamma power. The mean ± SEM of the residual power across the population is shown at the bottom. Cells with residual gamma power over 0.20 are below the black dotted line (44 cells). The vertical scale bar indicates 20 cells, and the green arrows point to the examples shown in (C). (E) We recorded activity in the LGN while presenting gray screen stimuli with light intensity changing every 4 s. From bottom: average spectrogram of spiking activity of a single neuron, triggered on the onset of four light intensities (1.3, 4.7, 16.9, and 55.4 cd/m^2^; each intensity was presented 30 times). The mean power spectrum for each intensity is shown on the top (ranging from light gray for 1.3 to black for 55.4). (F) Narrowband gamma power is positively correlated with light intensity (ρ = 0.74; p < 10^−21^; n = 15 recordings). Error bars show mean ± SEM. (G) The peak narrowband frequency is positively correlated with intensity (ρ = 0.67; p < 10^−16^; n = 15 recordings).

The power of the narrowband gamma oscillation varied strongly across the depth of visual cortex and was markedly higher in layer 4 (L4; Figures 3A, 3B, and S3). We recorded the activity across different laminae of V1 using a linear multi-electrode array (16 sites, 50 μm spacing; Figure 3A) and identified L4 in each recording as the location showing the earliest current sink (Mitzdorf, 1985) in response to a contrast-reversing checkerboard stimulus (Figure 3A). This layer also showed the strongest ∼60 Hz narrowband gamma peak (Figure 3B). The highest residual power was observed at the same depth as the largest current sink in all recordings (correlation ρ = 0.98; p < 10^−3^; n = 6 recordings; Figure S3).

Because L4 receives strong inputs from the LGN, we next asked if the narrowband gamma oscillation was also present in these inputs, and we found that it is evident in many LGN neurons (Figures 3C and 3D). We used silicon probes to record from 323 LGN neurons from 7 awake head-fixed mice viewing a uniform gray screen (∼50 cd/m^2^). Inspection of spike-train power spectra revealed that many neurons fired rhythmically at frequencies close to 60 Hz (Figures 3C and 3D). We quantified the rhythmicity of each neuron as the power in the narrowband gamma frequency range compared to a baseline prediction based on a smooth power spectrum (the “residual spectrum”; Figure S1). This measure yielded high values in a sizeable proportion of LGN neurons (Figure 3D). For instance, 14% of LGN neurons (44/323; n = 18 recordings from 7 animals) showed a narrowband gamma peak greater than 20% of the baseline prediction (dotted line in Figure 3D). Consistent with our results in V1 (Figure 2), the power of narrowband gamma in LGN decreased with increasing levels of contrast (ρ = −0.53; p < 10^−4^; n = 9 recordings; Figures S4A–S4C).

The narrowband gamma oscillation entrained the activity of many LGN neurons in a coherent fashion (Figure S5). We observed the narrowband oscillation in the spike time autocorrelations (Figure S5A) and found that it was coherent between simultaneously recorded pairs of neurons (0.33 ± 0.04, mean ± SD; n = 215 cell pairs where both cells had over 20% residual power), being clearly visible in both the cross-correlogram and coherence spectrum computed from the spike trains of simultaneously recorded pairs (Figures S5B and S5C). Narrowband gamma in LGN therefore represents a network-wide oscillation, rather than independent oscillations in individual cells. Individual LGN neurons had diverse but consistent phases with respect to the oscillation: computing each neuron’s preferred phase in two halves of the data yielded a similar estimate (correlation ρ = 0.69; p < 10^−6^; Figure S5D). The strength and the phase of narrowband gamma entrainment in LGN neurons bore no obvious relationship to their visual preferences, such as receptive field polarity and contrast sensitivity (data not shown).

### Narrowband Gamma Power and Frequency Increase with Light Intensity Levels

To investigate how narrowband gamma depends on light intensity, we recorded LGN neurons using extracellular multi-tetrode array recordings while varying the light intensity of a gray screen between ∼1 and ∼95 cd/m^2^ (Figures 3E–3G). The light intensity changed every 4 s (Figure 3E), presented in interleaved sequences. The narrowband oscillation was seen when light intensities were above 4 cd/m^2^, with power that increased with light intensity (ρ = 0.74; p < 10^−21^; n = 15 recordings; Figures 3E, 3F, and S4D). Light intensity also had an effect on the oscillation’s frequency, which increased from close to 50 Hz at low intensities (<5 cd/m^2^) to around 60 Hz at high intensities (>30 cd/m^2^). As a result, we found a strong correlation between the peak frequency of the narrowband gamma oscillation and the log light intensity level (ρ = 0.67; p < 10^−16^; n = 15 recordings; Figure 3G).

### Narrowband Gamma Is Increased in Active Animals

The strength of narrowband gamma oscillations was related not only to visual factors (light intensity and contrast) but also to the behavioral state of the animal. Consistent with previous reports (Lee et al., 2014, Niell and Stryker, 2010), we found that narrowband gamma power is highly correlated with running speed (correlation ρ = 0.49 ± 0.04, mean ± SEM; n = 10 recordings in V1). This observation, however, does not imply that behavioral state itself directly modulates gamma power. Indeed, since the mouse pupil dilates during locomotion (Erisken et al., 2014, McGinley et al., 2015a, McGinley et al., 2015b, Reimer et al., 2014, Vinck et al., 2015), and since narrowband gamma power depends on light intensity (Figure 3F), it remains possible that the correlation between locomotion and narrowband gamma reflects a greater amount of light striking the retina when pupils are dilated during running.

To answer this question, we measured gamma power after applying the anti-muscarinic agent tropicamide to the eye surface. This procedure caused full dilation of the pupil (Figure S6A), thus removing the correlation of pupil diameter with running speed. We found that the correlation of narrowband gamma power with running speed persisted even in these conditions of fixed pupil diameter (correlation ρ = 0.35; p < 0.01; Figure S6B), confirming that the correlation of narrowband gamma oscillation with locomotion does not simply reflect an indirect effect of increased light flux after pupil dilation.

### Narrowband Gamma Does Not Require V1 Activity

The presence of the narrowband gamma oscillation in LGN suggests, but does not prove, that the cortex inherits this rhythm from the thalamus. Indeed, this oscillation could be generated in V1 independently of LGN activity. To investigate this question, we recorded activity in V1 using extracellular multi-electrode arrays, while silencing the activity in the LGN by optogenetically activating thalamic reticular nucleus (TRN) neurons (Reinhold et al., 2015). Silencing LGN strongly suppressed LFP activity in V1 at all frequencies, including the narrowband gamma peak close to 60 Hz (Figures S7A–S7C). This result indicates that the narrowband gamma oscillation cannot be sustained in V1 without input from the thalamus.

**Figure 4:**
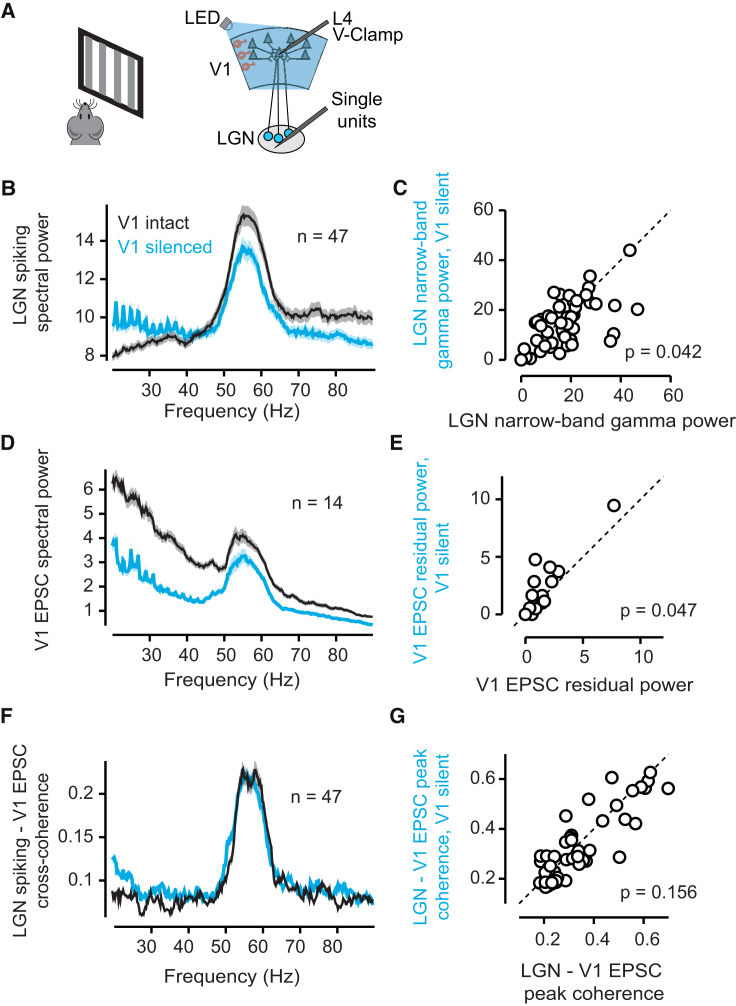
Narrowband Gamma Oscillations in LGN and V1 EPSCs Persist in the Absence of V1 Spiking (A) We made whole-cell voltage-clamp recordings of EPSCs in neurons in L4 of V1 while simultaneously recording single-unit activity in the LGN of VGAT-ChR2 mice under light urethane anesthesia. V1 could be silenced by shining a blue LED over the region. (B) Mean (±SEM) spectral power across LGN spike trains (n = 47 neurons) when V1 activity was intact (black) or silenced (cyan). (C) Comparison of the peak power of the narrowband gamma when cortical activity is intact or silenced (n = 47 neurons). (D) Mean (±SEM) EPSC spectral power across neurons with and without cortical silencing (n = 14 neurons). (E) Comparison of residual narrowband gamma power when cortical activity is intact or silenced (n = 14 neurons). (F) The cross-coherence between EPSCs recorded in V1 and LGN spiking activity shows a peak at the narrowband gamma frequency, which does not change with cortical silencing (n = 47 pairs). (G) Comparison of maximum LGN-V1 cross-coherence when cortical activity is intact or silenced (n = 47 neurons).

A second hypothesis is that the oscillation could rely on the presence of cortico-thalamic connections. To test this hypothesis, we asked whether silencing V1 would suppress the narrowband gamma oscillation in LGN and in the excitatory currents that it elicits in L4 cortical neurons. We performed these experiments in VGAT-ChR2 mice, where excitatory V1 activity could be silenced optogenetically across layers (Lien and Scanziani, 2013) (Figures S7D–S7I). To facilitate combined intracellular and extracellular recordings (Figure 4A), the mice were lightly anesthetized with urethane. This light anesthesia did not impede the narrowband gamma oscillation: LGN neurons showed rhythmic firing at frequencies close to 60 Hz, and EPSCs recorded simultaneously in L4 neurons showed a coherent oscillation (Figures 4B, 4D, and 4F).

Silencing the cortex optogenetically did not abolish the narrowband oscillation observed in LGN or the EPSCs in L4 neurons (Figures 4 and S7J–S7L). Silencing V1 caused no significant change in the frequency (p = 0.312; paired t test; n = 47 neurons with greater than 50% residual power in either condition; Figure 4B) and only a slight reduction of power (p = 0.042; Figure 4C) of the narrowband oscillation seen in LGN spike trains. While cortical silencing reduced L4 EPSC spectral power at all frequencies, there was actually a slight increase in the residual power of the narrowband gamma oscillation (p = 0.047; n = 14; Figures 4D, 4E, and S7L), consistent with addition of a narrowband oscillation inherited from the thalamus, to cortically generated broadband activity. The cross-coherence between LGN spiking activity and the EPSCs in L4 V1 neurons showed a clear peak at the narrowband gamma frequencies, which was unaffected by cortical silencing (p = 0.15; n = 47; Figures 4F and 4G). These results demonstrate that the narrowband gamma oscillation in LGN persists in the absence of feedback from V1.

## Discussion

We have shown that the narrowband gamma oscillation in mouse V1 has several major differences from the more commonly described broadband gamma rhythm. The power of narrow- and broadband gamma activity was uncorrelated, and visual contrast suppressed the narrowband gamma oscillation while it increased broadband gamma rhythms. Within cortex, the narrowband gamma oscillation was strongest in L4, and cortical intracellular recordings revealed narrowband gamma in excitatory, but not inhibitory, synaptic currents. Narrowband gamma oscillations persisted in LGN ensembles and L4 synaptic inputs, even after silencing of cortex.

We therefore conclude that the narrowband gamma oscillation (2–5 Hz bandwidth oscillation close to 60 Hz) is generated in thalamus and possibly even retina, representing an entirely different phenomenon than broadband gamma (30–90 Hz rhythms). Whereas broadband gamma rhythms involve cortical inhibitory networks (Cardin et al., 2009, Penttonen et al., 1998, Sohal et al., 2009, Wang and Buzsáki, 1996), narrowband gamma is transmitted to cortex by the rhythmic firing of thalamic ensembles. Although resonance in thalamocortical loops has been proposed as a mechanism for the generation of high-frequency oscillations (Steriade, 1997, Steriade, 2000), we found that the narrowband gamma oscillation was transmitted from LGN to visual cortex even after cortical firing was abolished optogenetically. We therefore propose that the oscillation is generated either within thalamic circuits or by their interactions with their retinal inputs.

Our results are consistent with previous reports of narrowband oscillations in mouse visual cortex at frequencies close to 60 Hz (Lee et al., 2014, Niell and Stryker, 2010). Furthermore, the oscillation we study here might be homologous to an oscillation previously described in LGN of anesthetized cats (Castelo-Branco et al., 1998, Koepsell et al., 2009, Koepsell et al., 2010, Neuenschwander and Singer, 1996), which showed extremely narrowband rhythmicity within single recordings. The frequency of that oscillation, however, varied between recordings, spanning a range of 45–114 Hz. In these cat recordings, the thalamic oscillation was highly synchronous with a retinal oscillation determined either by simultaneous recording or by analysis of intracellular excitatory postsynaptic potentials (EPSPs) in thalamus. If this proposed homology is correct, it would suggest that the narrowband gamma recorded here might reflect an oscillation generated in retina and transmitted through LGN to visual cortex. The fact that the power of this oscillation is increased by locomotion (as well as by stimulation of mesencephalic locomotor nuclei at levels too small to evoke locomotion; Lee et al., 2014) suggests that its amplitude is likely to be modulated by thalamic circuitry.

What function might this narrowband gamma oscillation play in visual processing? Although a definitive answer to this question requires further work, the present data are sufficient to formulate two hypotheses.

A first hypothesis is that the narrowband gamma oscillation represents an “idling” rhythm. In this interpretation, narrowband gamma represents a default state of LGN activity when visual processing is not being carried out, similar to the proposed function of the visual alpha rhythm and the beta oscillation of the motor system. This hypothesis would explain why this oscillation is suppressed by increases in visual contrast.

In the second hypothesis, the narrow- and broadband gamma oscillations would represent specific channels for thalamocortical and corticocortical communication. It has been proposed that oscillatory patterns with different frequency characteristics may allow routing of information between specific brain structures. For example, the coherence of slow gamma between hippocampal areas CA1 and CA3, and fast gamma between CA1 and entorhinal cortex (Colgin et al., 2009), may indicate the existence of two separate transmission channels in the hippocampal formation. Analogously, the narrowband gamma oscillation may provide a specific channel for feedforward transmission from LGN to visual cortex, while broadband gamma—which is generated in cortical regions—enables transmission of information between cortical circuits.

## Experimental Procedures

Please see the Supplemental Experimental Procedures for details of all methods.

## Author Contributions

Conceptualization, A.B.S., M.K., M.C., and K.D.H.; Methodology, A.B.S., A.D.L., B.H., M.R.R., A.A., K.R., and L.B. (specific experimental contributions are listed in Table S1); Formal Analysis, A.B.S.; Writing – Original Draft, A.B.S., M.C., and K.D.H.; Writing – Review & Editing, all authors. M.C. and K.D.H. are co-senior authors.

## Supplemental Information

Document S1. Supplemental Experimental Procedures, Figures S1–S7, and Table S1

Document S2. Article plus Supplemental Information
