# Supplementary material for: Subcortical Source and Modulation of the Narrowband Gamma Oscillation in Mouse Visual Cortex
Source: Neuron. 2017 Jan 18;93(2):315–22. doi: 10.1016/j.neuron.2016.12.028 (PMC5263254; doi:10.1016/j.neuron.2016.12.028)
Supplement: Document S1. Supplemental Experimental Procedures, Figures S1–S7, and Table S1 [file mmc1.pdf]

Neuron, Volume *93*

Supplemental Information

Subcortical Source and Modulation of the Narrowband Gamma Oscillation in Mouse Visual Cortex

Aman B. Saleem, Anthony D. Lien, Michael Krumin, Bilal Haider, Miroslav Román Rosón, Asli Ayaz, Kimberly Reinhold, Laura Busse, Matteo Carandini, and Kenneth D. Harris

Supplementary Figures

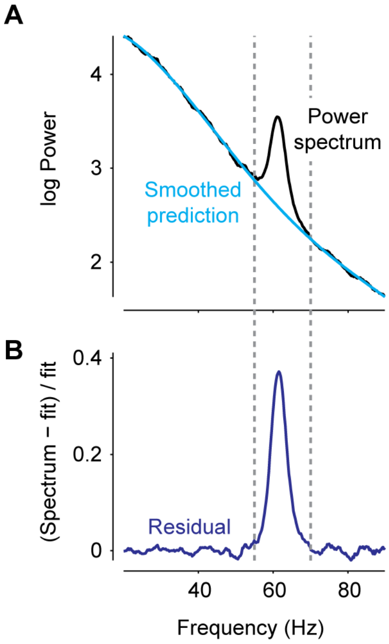

Figure S1 (related to Figure 1): Analysis of narrow-band oscillation. A. Example power spectrum from one recording session (Black). The power spectrum (excluding the narrow-band gamma region highlighted by the dotted lines) is fitted by a 4th order polynomial (smoothed prediction, cyan). **B.** The residual spectrum is the difference between the actual data and the fitted function, divided by the fitted function. The peak frequency of narrow-band gamma was calculated as the position of the peak in the residual spectrum.

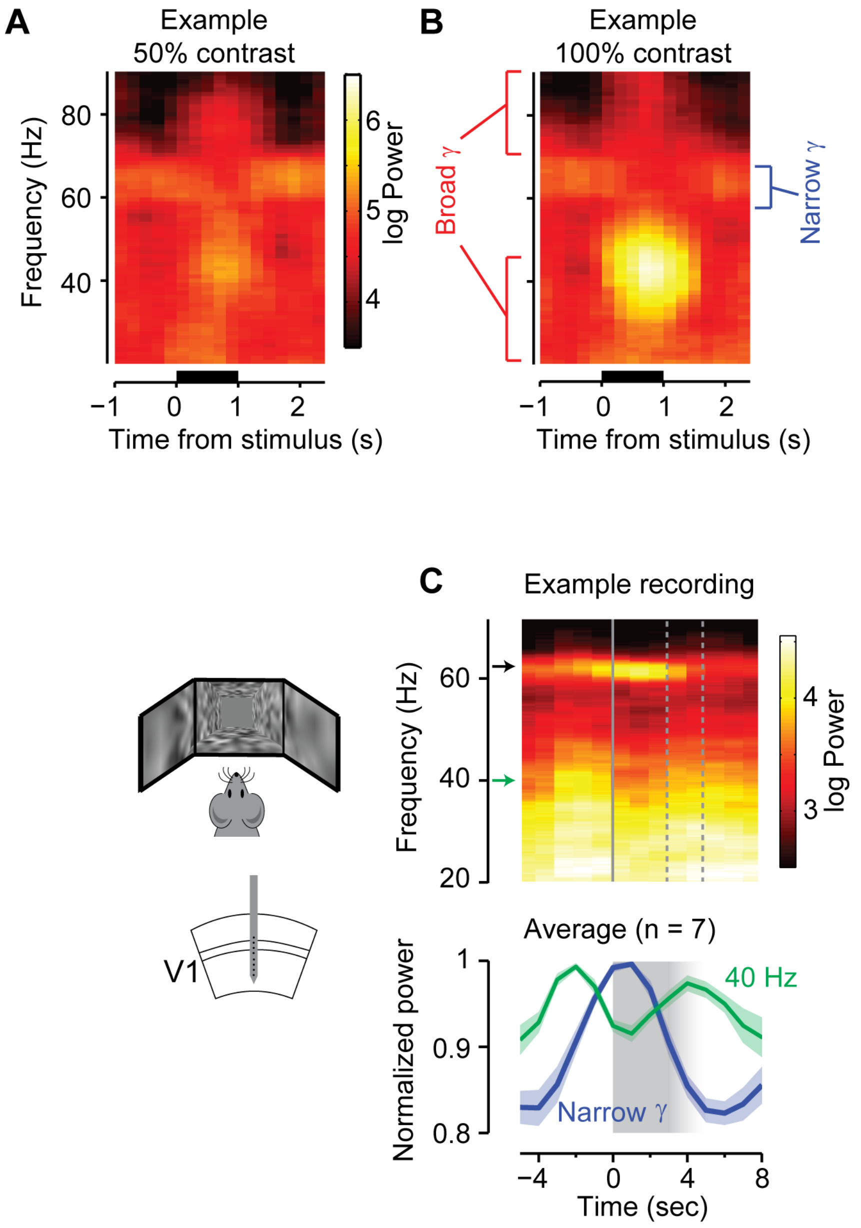

Figure S2 (related to Figure 2): Broadband gamma rhythms are enhanced by visual contrast while the narrowband gamma oscillation is suppressed. A-B. Average LFP spectrogram triggered on presentation of a drifting grating stimulus at 50% contrast (**A**) and 100% contrast (**B**), in a single experiment. Bars below indicate the period of presentation of a 50° sinusoidal drifting grating in the retinotopic location of the recorded area. **C.** Average LFP spectrogram triggered on the onset of a gray screen period in a virtual reality environment. The gray screen period lasted between 3-5 secs after which the animal re-enters the high contrast virtual environment. **(Bottom)** normalized power (power / maximal power) at the narrow band frequency (Narrowband gamma; Blue) and at 40 Hz (Green). The shaded regions show the mean ± SEM across 7 recording sessions. The gray shaded region shows the period of gray screen presentation, which varied between 2 to 4 s. Arrows in **C** indicate the frequencies shown in the bottom plots.

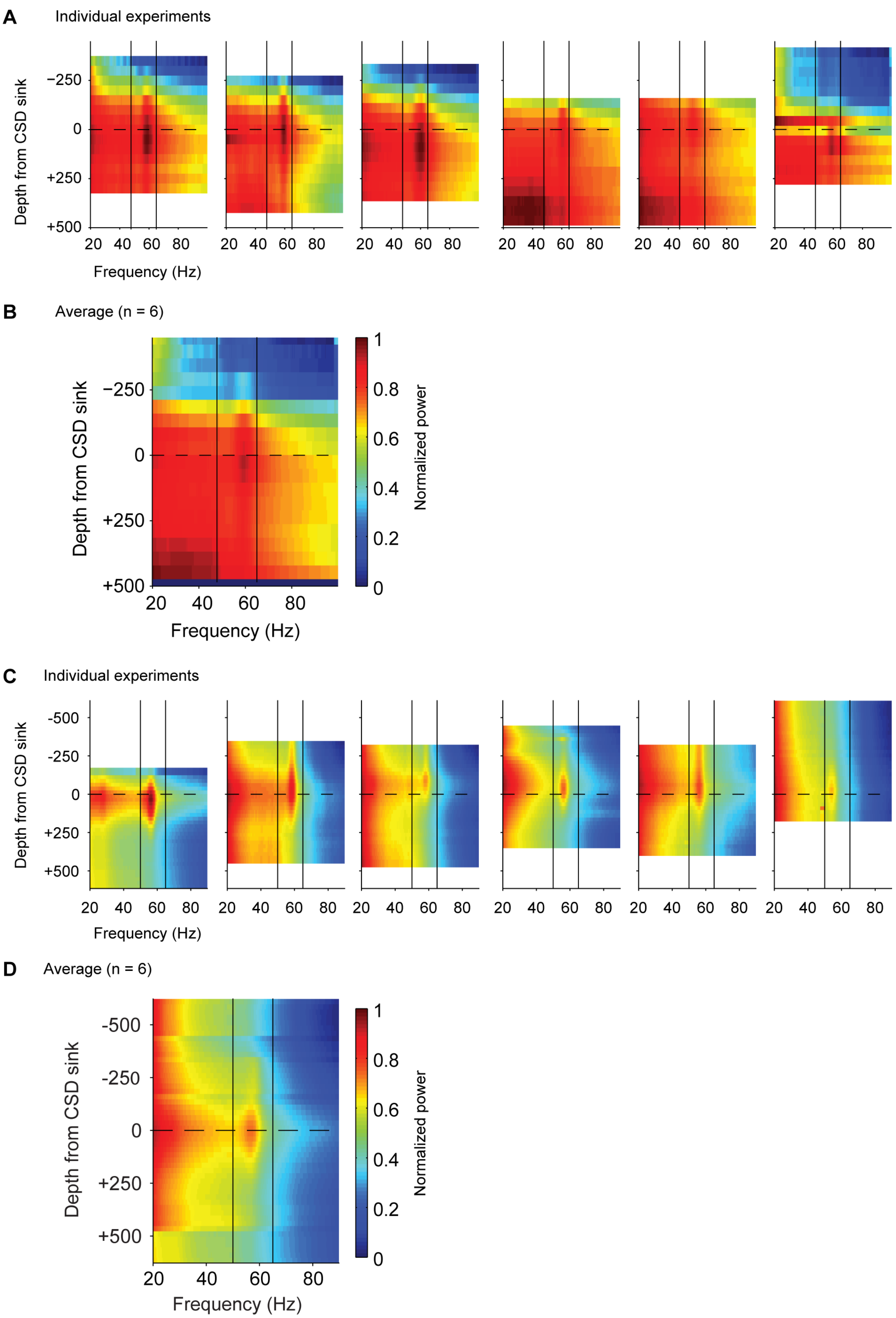

Figure S3 (related to Figure 3): Narrowband gamma is highest in Layer 4 of V1 across experiments. A,C. The power across frequencies as a function of depth from the putative Layer 4 (peak of the CSD sink) across six experiments. The vertical lines mark the frequency range (50-65Hz) of narrow-band

gamma oscillation. **B, D.** The depth profile of narrow band gamma averaged across all experiments. Experiments in A-B were conducted using protocol A and experiments in C-D were conducted using protocol B. We thank R. Dulinskas for help with data collection (C, D).

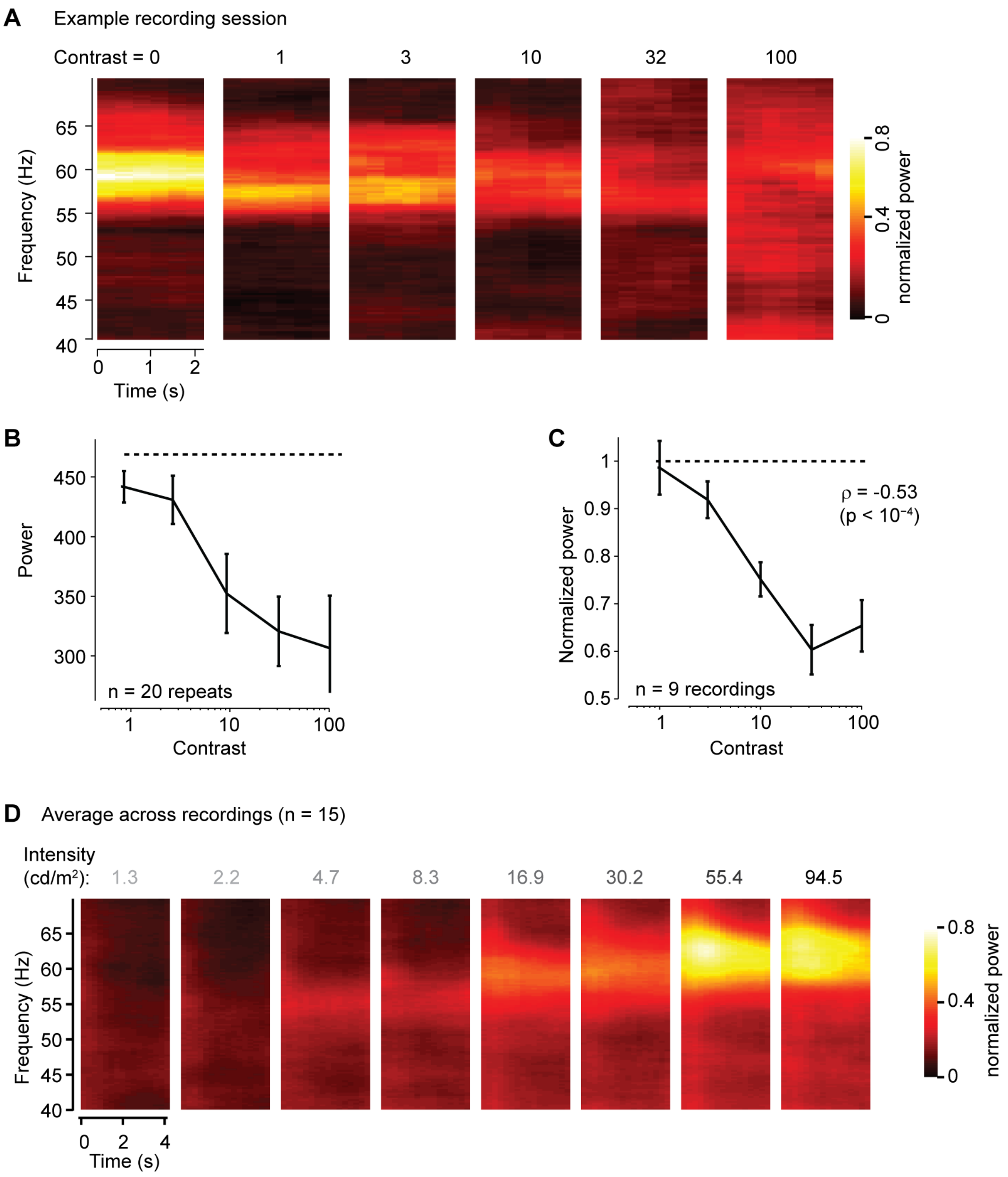

Figure S4 (related to Figures 2 & 3): Narrowband gamma oscillation in LGN spiking is suppressed by contrast. A. Average spectrogram of spiking activity of a single neuron, triggered on the start of full-screen grating stimuli (0%, 1%, 3%, 10%, 32% & 100% contrast, each contrast level repeated 20 times). The stimulus was presented for 2s, with a 3s inter-stimulus interval with 0% contrast (iso-luminant gray screen). **B.** Narrowband power decreases as a function of the contrast presented for the recording session shown in **A**. Errorbars show mean ± SEM. The dotted line shows the mean narrowband power at 0% contrast. **C.** Same as B, averaged across all the recording sessions (ρ = -0.53, p<10^{-4}; n = 9 recordings). The peak narrowband power was normalized for each recording based on the power at 0% contrast. **D.** Average spectrogram of spiking activity across all recording sessions for the 8 different intensities presented (similar to Figure 3E), showing an increase in gamma power with intensity (n = 15 recordings). The spectrograms of each recording session were normalized by the maximum average power in each experiment, before averaging across recordings.

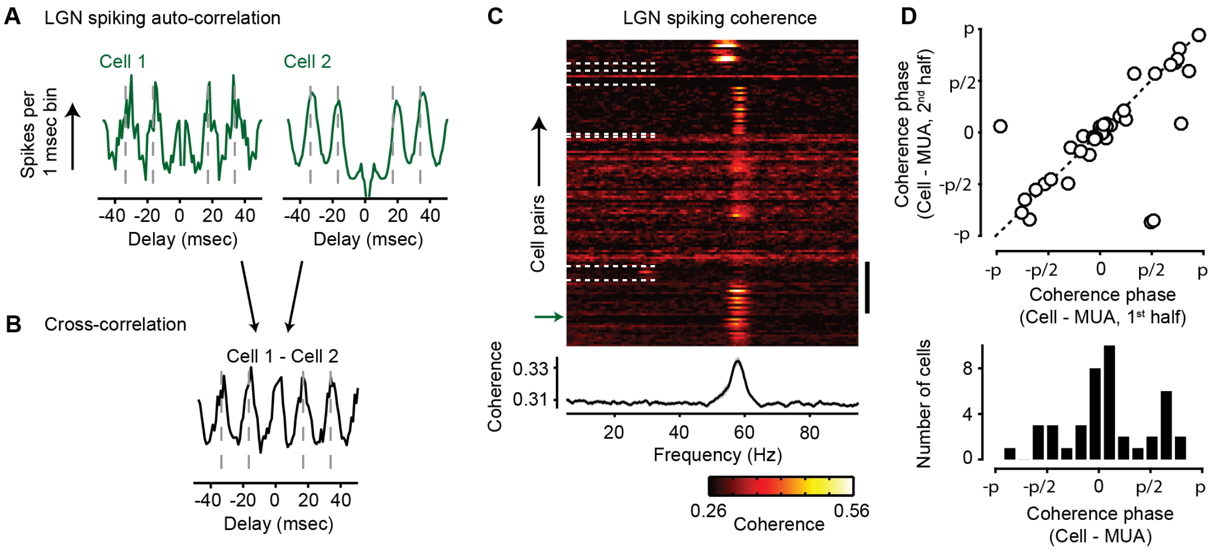

Figure S5 (related to Figure 3): Narrowband gamma is coherent between LGN units. A. Two examples of the spike auto-correlation of LGN neurons (same cells as C), showing a clear oscillation at narrowband gamma frequency. Dashed lines indicate the positions of the peaks expected for a 60 Hz modulation (at ±16.7 and ±33.4 msec). **B.** The cross-correlation of spike times of neurons in G. **C.** Coherence spectra for all pairs of simultaneously recorded neurons with residual gamma power greater than 0.20 (n = 130 pairs). White dotted lines separate pairs from different recording sessions. The mean ± SEM of the coherence spectra across all cell pairs is shown at the bottom. The scale bar indicates 20 cell pairs, and the green arrow points to the example cell pair shown in panels F. **D.** (Top) Preferred phase of each oscillatory cell relative to the population of other simultaneously recorded neurons, calculated in the first and second halves of the recording. (Bottom) The distribution of the preferred phase across cells (n = 44 cells).

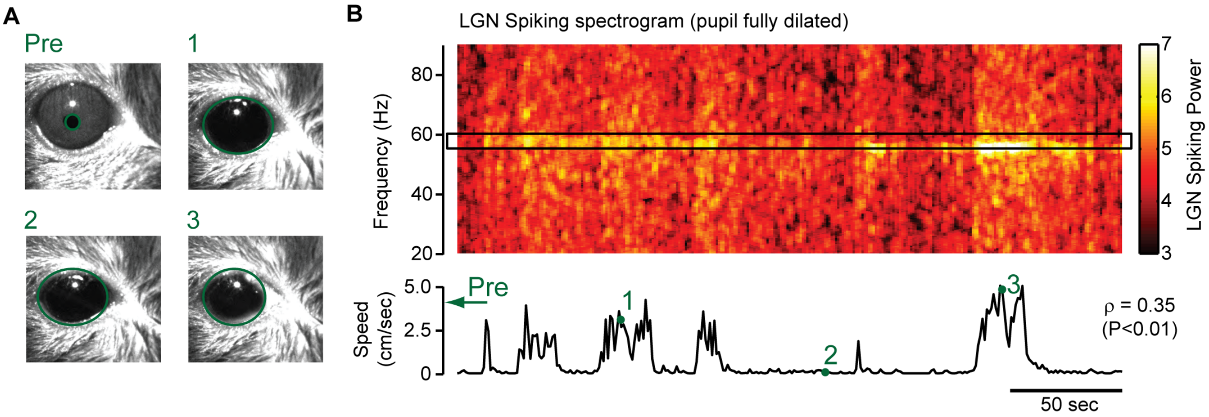

Figure S6 (related to Figure 3): Narrowband gamma power is correlated with running even with a fully dilated pupil. A. Images of the eye before dilation (Pre), and after being dilated (Image 1-3) with Tropicamide (>10 mins after full dilation), when the animal was stationary (Image 2) or running (Images 1 and 3). The green ovals over the images indicate the detected pupil area. **B.** Spectrogram of LGN spiking activity at different frequencies as a function of time. The trace below shows the running speed of the animal. Narrowband gamma power (mean in region highlighted by box) was positively correlated with running speed (ρ = 0.35, $p<0.01$). The time points of the images are shown in **A** are marked with green numerals.

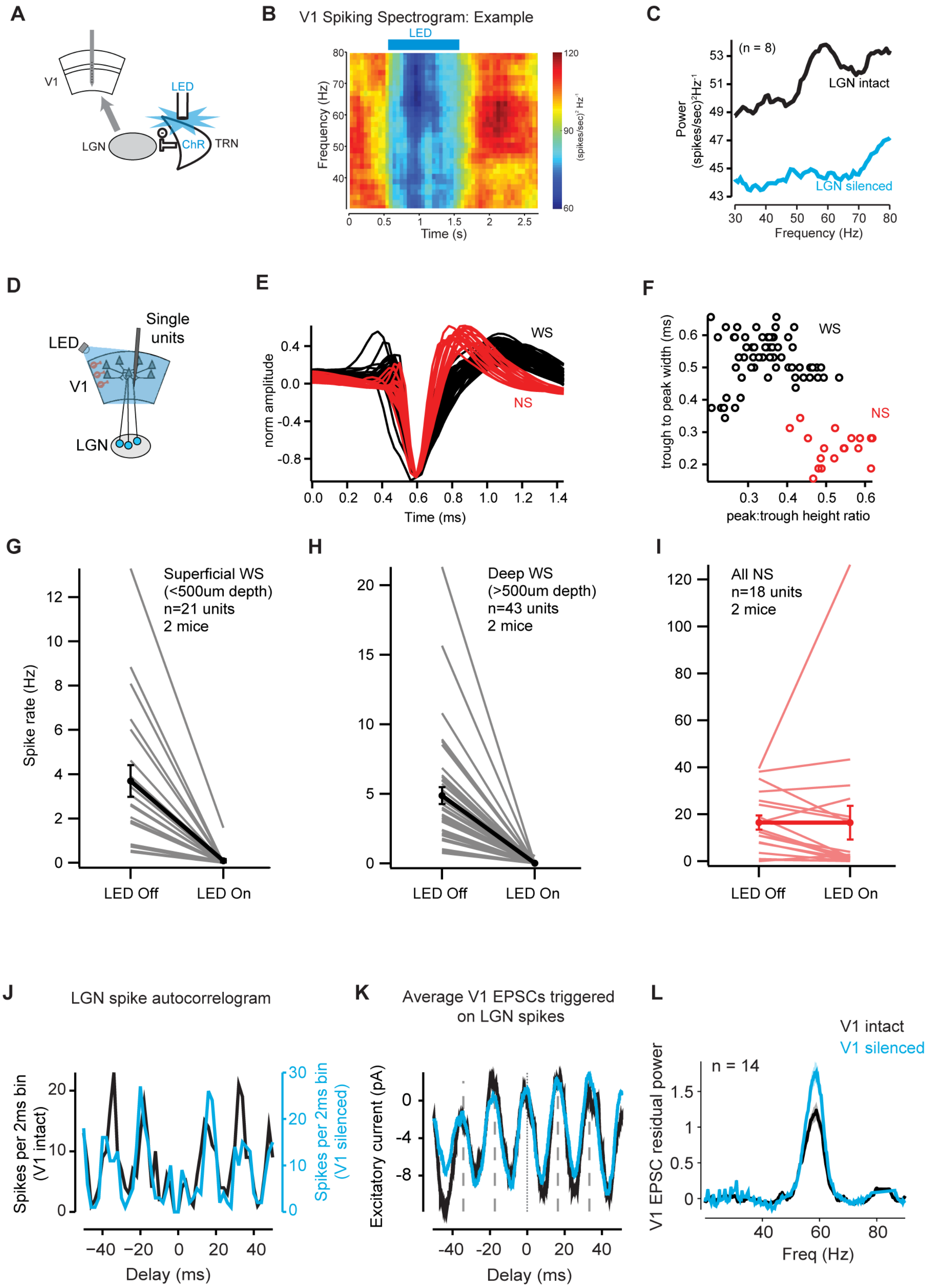

Figure S7 (related to Figure 4): Narrowband gamma oscillation in V1 is abolished by LGN inactivation, but not by V1 inactivation. A. We recorded activity in V1 using a multi-electrode array (in awake mice), while inactivating the LGN by activating the TRN using an LED. **B.** Spiking spectrogram of V1 activity triggered on LED stimulation (Example experiment) **C.** The average power of V1 during (cyan) and after (black) LGN inactivation (n = 8 experiments). The narrowband gamma

peak is abolished, together with a decrease in power at all frequencies. **D.** We recorded single-unit activity in the V1 of VGAT-ChR2 mice under light urethane anesthesia, where V1 could be silenced by shining a blue LED over the region. **E.** The plot shows superimposed waveforms of V1 neurons, color coded based on whether they were classified as wide spiking (WS, black) or narrow spiking (NS, red). **F.** The neurons were classified as WS or NS based on the peak-trough height ratio and trough to peak width (based on methods from Niell & Stryker, 2008). **G.** Turning on the LED suppressed the firing of all the WS neurons recorded from superficial layers (<500um; n = 21 neurons). **H.** Turning on the LED suppressed the firing of all the WS neurons recorded from deep layers (>500um; n = 43 neurons). **I.** The activity of NS neurons did not change significantly between the LED On and LED Off conditions (all depths; n = 18 neurons). **J.** Spike time autocorrelation of an LGN single unit when V1 activity was intact (black) or silenced (cyan) **K.** Average EPSCs recorded by voltage-clamping a V1 layer 4 neuron at -70mV, triggered on spikes of an LGN neuron (same neuron as in B, D), when V1 activity was intact (black) or silenced (cyan). Excitatory current negative. **L.** Mean (± SEM) residual power of the EPSC spectral power across neurons without and without cortical silencing (n = 14 neurons; related to Figure 4D).

Table S1 (related to Figure 1-4): Recording list. List of animal strains, recording electrodes, stimuli and data analyses parameters

Figure number	Animal strain (number of recordings)	Sex and number of animals	Electrodes	Stimuli	Parameters for analysis (Chronux)	Experimenter (Protocol followed)
1A,D	PV-Cre x Ai32 (2 recordings, 1 example shown)	2M	Neuronexus A2x4 tet	Flickering LED	Tapers: 5,9 Window: 3s, 1s shift	ABS (A)
1B,C	C57BL6 (1 example recording)	1M	Neuronexus A1x16	Gray screen / Dark	Tapers: 5,9 Window: 3s, 1s shift	ABS (A)
1E	C57BL6 (11 cells)	9F	Glass electrodes	Gray screen	Tapers: 3,2 Window: 0.33s, 0.5s shift	BH (A)
2A-D	C57BL6 (7 recordings)	3M	Neuronexus A1x16	Contrast, grating (2cycles/s, 0.05cycles/^{0})	Tapers: 5,9 Window: 1s, 0.2s shift	AA & ABS (A)
2E	C57BL6 (9 recordings)	3M	Neuronexus A1x32Edge or A2x4 tet	Virtual reality	Tapers: 5,9 Window: 3s, 1s shift	ABS (A)
3A-B	C57BL6 (6 recordings)	3M	Neuronexus A1x16	Contrast reversing checkerboard (3A; 15^0 squares, 2 cycles/s) or gray screen (3B)	Tapers: 5,9 Window: 3s, 1s shift	ABS & AA (A)
3C-D	C57BL6 (18 recordings)	2M / 5F	Neuronexus A1x32Edge	Gray screen	Tapers: 5,9 Window: 3s, 1s shift	MRR (B)
3E-F	C57BL6 (15 recordings)	3F	Neuronexus A2x4 tet	Full-field intensity & contrast	Tapers: 5,9 Window: 3s, 1s shift	ABS (A)
4	VGAT-ChR2 (14 whole-cell & 12 LGN recordings)	12 (M/F)	Glass electrodes + Neuronexus Buzsaki32	Full-field moving grating	Tapers: 3,5 Window: 2.5s	TL (C)
S1	C57BL6 (1 recording)	1M	Neuronexus A2x4 tet	Virtual reality	Tapers: 5,9 Window: 3s, 1s shift	ABS (A)
S2A, B	Same as Figure 2					
S2C	C57BL6 (7 recordings)	4M	Neuronexus A1x32Edge	Virtual reality	Tapers: 5,9 Window: 3s, 1s shift	ABS (A)
S3A, B	Same as Figure 3A-B					
S3C, D	C57BL6 (6 recording)	2 M	Neuronexus A1x32 (sites 25µm apart)	Contrast reversing checkerboard (3A; 15^0 squares, 2 cycles/s) or gray screen (3B)	Tapers: 5,9 Window: 3s, 1s shift	RD (B)
S4	Same as Figure 3C-D					
S5	Same as Figure 3E-F					
S6	C57BL6 (1 recordings)	1M	Neuronexus A2x4 tet	Gray screen	Tapers: 5,9 Window: 2.3s, 1s shift	ABS (A)
S7A-C	GAT2-Cre (8 recordings)	8 (M/F)	Neuronexus Buzsaki16	Full-field moving grating	Tapers: 3, 5 or 10; Window: 1s, 0.05s shift	KR (C)
S7E-I	VGAT-ChR2	2 (M/F)	Neuronexus Buzsaki32	Full-field moving grating	N/A	TL (C)
S7J-L	Same as Figure 4					

Experimental procedures

Experimental methods

The experiments were performed in three different laboratories, following one of three protocols below. We list the protocol followed for each experiment in Supplementary Table 1.

Protocol A: All procedures were conducted in accordance with the UK Animals Scientific Procedures Act (1986). Experiments were performed at University College London under personal and project licenses released by the Home Office following appropriate ethics review. We used both male and female mice of two strains: C57BL6 (17 animals) and PV-Ai32 (2 animals; RRID:IMSR_JAX:008069 / RRID:IMSR_JAX:012569) (full listing in Supplementary Table 1). Experimental methods are described in detail in Ayaz et al. 2013, Haider et al. 2013 and Saleem et al. 2013(Ayaz et al.,2013; Haider et al.,2013; Saleem et al.,2013). Briefly, animals were chronically implanted with a custom-built head post and recording chamber (3-4 mm inner diameter) under isoflurane anesthesia. They were then allowed to recover for 3 days with oral analgesic Rimadyl. Following recovery, the animals were acclimatized for 3-4 days to the head fixation apparatus. A craniotomy was performed between 4 – 24 hrs prior to the first recording session. Whole-cell patch-clamp recordings of EPSCs and IPSCs were performed with a cesium-based internal solution, with QX-314 (0.5mM) and tetraethylammonium (TEA) (5mM) included to block voltage-gated Na+ and K+ conductances. Extracellular spikes were isolated using the KlustaView Suite(Rossant et al.,2016). Visual stimuli were presented on LCD monitors that were gamma corrected. For most experiments, the mean light intensity level presented on the monitors was ~50 cd/m^2. The experiments in the dark were conducted making sure that all non-essential equipment was turned off, and essential equipment producing any light were covered with a filter (787 Marius Red; LEE filters) that only allows light that is not visible to mice. The light intensity level under these conditions was <10^{-2} cd/m^2, below the sensitivity of the light meter. For experiments in Figure 5 and Supplementary Figure S6, we placed Fresnel lenses in front of the three monitors to ensure that the light intensity entering the eye is independent of the viewing angle on the LCD monitors. With Fresnel lenses the light intensity level could vary from ~0.2 cd/m^2 to ~95 cd/m^2.

Protocol B: All procedures were performed on awake, adult mice and complied with the European Communities Council Directive 2010/63/EC and the German Law for Protection of Animals. All procedures were approved by the local authorities following appropriate ethics review. We used both male and female C57BL6 mice (full listing in Supplementary Table 1). Experimental methods are described in detail in Erisken et al, 2015 (Erisken et al.,2014). Briefly, animals were chronically implanted with a custom-built head post and recording chamber (3-4 mm inner diameter) under isoflurane anesthesia. Animals were then allowed to recover for a minimum of 3 days during which they received analgesics (Carprofen, 5 mg/kg, s.c.) and antibiotics (Baytril, 5 mg/kg, s.c.). Following complete recovery, the animals were handled and acclimatized for a minimum of ~1 week to head fixation and the spherical treadmill. One day before the first recording session, a craniotomy was performed under isoflurane anesthesia. Spikes were isolated using the Klusters suite (Hazan et al.,2006) in combination with KlustaKwik (Schmitzer-Torbert et al.,2005). Visual stimuli were presented on LCD monitors that were gamma corrected. For most experiments, the mean light intensity level presented on the monitors was ~50 Cd/m^2.

Protocol C: All procedures were conducted in accordance with the National Institutes of Health guidelines and with the approval of the Committee on Animal Care at UCSD (protocol S02160M). Animals were housed on a reverse light cycle in cages of four mice or less. At the time of electrophysiology, all animals were older than 3.5 weeks. Both male and female animals were used in an approximately equal ratio.

Experimental methods are described in detail in Lien et al. 2013 and Reinhold et al. 2015 (Lien and Scanziani,2013; Reinhold et al.,2015). Briefly, simultaneous V1 L4 intracellular whole-cell and LGN extracellular (Buzsaki32 probe, Neuronexus) recordings were performed in VGAT-ChR2 mice (JAX 014548 Zhao, et al; RRID:IMSR_JAX:014548) under urethane (1.5g/kg, IP)/chlorprothixene (2-4mg/kg, IP) anesthesia. V1 neurons were held in voltage clamp at -70 mV to record excitatory currents. Full field drifting bar gratings (100% contrast, 2 Hz temporal frequency, 0.04 cycles/deg spatial frequency) were presented on a monitor in the right visual field for 2.3 s preceded by 0.7 s of a gray screen of mean light intensity . Optogenetic suppression of V1 was achieved by constant illumination of V1 with blue light from a 1mm fiber-coupled LED positioned several millimeters over the crantiotomy (455 nm, 20mW at the fiber tip, Doric) on interleaved trials starting 645 ms prior to grating onset and extinguishing after grating offset. For experiments inactivating the LGN, V1 extracellular (Buzsaki16 probe, Neuronexus) recordings were performed in awake Gad2-Cre mice (Jackson Labs stock number: 010802 (Taniguchi et al.,2011); RRID:IMSR_JAX:010802) expressing Cre-dependent ChR2 (AAV2/1.CAGGS.flex.ChR2.tdTomato.SV40, Addgene 18917 (Boyden et al.,2005), from the University of Pennsylvania viral vector core) stereotactically confined to the thalamic reticular nucleus (TRN). Mice were head-fixed but free to spontaneously run or rest on a circular treadmill. Full field drifting bar gratings (100% contrast, 2 Hz temporal frequency, 0.04 cycles/deg spatial frequency) were presented on a monitor in the right visual field for 3 s preceded by 2.5 s of a gray screen of mean light intensity . Optogenetic suppression of LGN was achieved by constant illumination of TRN with blue light from a 473 nm laser coupled to an optical fiber of diameter 200 microns, the tip of which was positioned just above the TRN (stereotactic coordinates of tip: [1,540 µm posterior, 2,235 µm lateral, 3,158 µm ventral to bregma], 10mW at the fiber tip). Laser illumination of the TRN on interleaved trials starting 0.2 ms after grating onset and lasting 1 second led to a 70% suppression (Reinhold et al.,2015) of spiking in LGN over the duration of illumination. Single units from both LGN and V1 recordings were isolated using UltraMegaSort (Hill et al.,2011). Visual stimuli were presented on an LCD monitor that was gamma corrected. The mean light intensity level presented on the monitors was 75 Cd/m^2 for experiments in Figure 4.

Analyses

Spectral Analysis

We used the Chronux toolbox (http://chronux.org/), which is based on multi-taper methods, for spectral analyses. We measured the spectrogram of the local field potential using the function *mtspecgramc*. The power spectrum was calculated as the mean spectrogram across time. For spiking spectra, we used *mtspecgrampt* for the spike times (measured at 1 msec temporal resolution). We list the parameters we used for the spectral analysis (number of tapers and temporal window) in Supplementary Table 1. To calculate coherence across cells pairs, we used the Chronux function *mtcohgrampt*. To measure phase differences for any cell, we calculated its coherence with the pooled spike times from all other units. We calculated phase as the angle of the mean joint power across time. For Figure 5 and Supplementary Figure S6, we pooled the spike times across all units

detected on a tetrode to calculate the spiking spectrogram. There were a total of 12 recording sessions in three animals where we recorded responses to varying levels of light intensity, two of these sessions had more than one tetrode in the LGN. There were a total of 8 recording sessions where we recorded responses to varying levels of contrast, one of which had more than one tetrode in the LGN.

Residual power and peak frequency

We characterised the narrowband frequency using the residual power spectrum: the fractional increase in the power of the spectrum compared to a smoothed prediction (Supplementary Figure S1). We first calculated the smoothed prediction by fitting a 4th order polynomial to the spectrum in the range 20-55 Hz and 70-90 Hz. This prediction generally captured the 1/f fall-off and the broadband gamma peak. We next calculated the residual spectrum at each frequency as:

$$Residual\ spectrum = \frac{Spectrum - Prediction}{Prediction}$$

We define the narrowband frequency of any recording as the frequency with the maximum power of the residual spectrum in the range 55-70Hz. For spiking spectra from the LGN, we refer to the peak value of residual spectrum as the 'residual gamma power'.

To statistically compare peak narrowband gamma frequencies between different LED flicker rates (Fig. 1D), we divided the recordings for each flicker rate into 5 equal subsets, computed a peak frequency in each subset, and compared these using paired t-tests for each pair of flicker rates.

Comodulation

To access how the power of different frequency components of the local field potentials varied together, we calculated the comodulation: the correlation of the spectral power of the two frequencies across time. We measured this at all pairs of frequencies in the range of 20-90 Hz.

Cross-correlation

When computing cross-correlograms for neurons from the same recording tetrode (Fig. 3F), the central bin was discarded and interpolated to avoid spike collision artifacts.

Supplementary references

Ayaz, A., Saleem, A.B., Scholvinck, M.L., and Carandini, M. (2013). Locomotion controls spatial integration in mouse visual cortex. Curr Biol 23, 890-894.
Boyden, E.S., Zhang, F., Bamberg, E., Nagel, G., and Deisseroth, K. (2005). Millisecond-timescale, genetically targeted optical control of neural activity. Nat Neurosci 8, 1263-1268.
Erisken, S., Vaiceliunaite, A., Jurjut, O., Fiorini, M., Katzner, S., and Busse, L. (2014). Effects of locomotion extend throughout the mouse early visual system. Curr Biol 24, 2899-2907.
Haider, B., Hausser, M., and Carandini, M. (2013). Inhibition dominates sensory responses in the awake cortex. Nature 493, 97-100.
Hazan, L., Zugaro, M., and Buzsaki, G. (2006). Klusters, NeuroScope, NDManager: a free software suite for neurophysiological data processing and visualization. Journal of neuroscience methods 155, 207-216.
Hill, D.N., Mehta, S.B., and Kleinfeld, D. (2011). Quality metrics to accompany spike sorting of extracellular signals. J Neurosci 31, 8699-8705.

Lien, A.D., and Scanziani, M. (2013). Tuned thalamic excitation is amplified by visual cortical circuits. Nat Neurosci 16, 1315-1323.

Reinhold, K., Lien, A.D., and Scanziani, M. (2015). Distinct recurrent versus afferent dynamics in cortical visual processing. Nat Neurosci 18, 1789-1797.

Rossant, C., Kadir, S.N., Goodman, D.F., Schulman, J., Hunter, M.L., Saleem, A.B., Grosmark, A., Belluscio, M., Denfield, G.H., Ecker, A.S., *et al.* (2016). Spike sorting for large, dense electrode arrays. Nat Neurosci 19, 634-641.

Saleem, A.B., Ayaz, A., Jeffery, K.J., Harris, K.D., and Carandini, M. (2013). Integration of visual motion and locomotion in mouse visual cortex. Nat Neurosci 16, 1864-1869.

Schmitzer-Torbert, N., Jackson, J., Henze, D., Harris, K., and Redish, A.D. (2005). Quantitative measures of cluster quality for use in extracellular recordings. Neuroscience 131, 1-11.

Taniguchi, H., He, M., Wu, P., Kim, S., Paik, R., Sugino, K., Kvitsiani, D., Fu, Y., Lu, J., Lin, Y., *et al.* (2011). A resource of Cre driver lines for genetic targeting of GABAergic neurons in cerebral cortex. Neuron 71, 995-1013.
