# Supplementary material for: Subcortical Source and Modulation of the Narrowband Gamma Oscillation in Mouse Visual Cortex
Source: Neuron. 2017 Jan 18;93(2):315–22. doi: 10.1016/j.neuron.2016.12.028 (PMC5263254; doi:10.1016/j.neuron.2016.12.028)
Supplement: Document S2. Article plus Supplemental Information [file mmc2.pdf]

Report

Neuron

Subcortical Source and Modulation of the Narrowband Gamma Oscillation in Mouse Visual Cortex

Highlights

- Mouse V1 exhibits a pronounced narrowband gamma oscillation close to 60 Hz
- This oscillation is strongest in layer 4 and specific to excitatory currents
- It increases with arousal and light intensity and decreases with visual contrast
- It is seen in lateral geniculate neurons, regardless of V1 activity

Authors

Aman B. Saleem, Anthony D. Lien, Michael Krumin, ..., Laura Busse, Matteo Carandini, Kenneth D. Harris

Correspondence

aman.saleem@ucl.ac.uk (A.B.S.),
m.carandini@ucl.ac.uk (M.C.),
kenneth.harris@ucl.ac.uk (K.D.H.)

In Brief

Saleem et al. discover that the narrowband gamma oscillation close to 60 Hz prevalent in the mouse visual cortex is inherited from the visual thalamus. The oscillation is enhanced by arousal and light intensity, and suppressed by visual contrast.

Saleem et al., 2017, Neuron *93*, 315–322
January 18, 2017 © 2017 The Authors. Published by Elsevier Inc.
http://dx.doi.org/10.1016/j.neuron.2016.12.028

Subcortical Source and Modulation of the Narrowband Gamma Oscillation in Mouse Visual Cortex

Aman B. Saleem,[1,2,3,7,11,*] Anthony D. Lien,[4] Michael Krumin,[1] Bilal Haider,[1,8] Miroslav Román Rosón,[5,6] Asli Ayaz,[1,9] Kimberly Reinhold,[4] Laura Busse,[5,10] Matteo Carandini,[1,*] and Kenneth D. Harris[2,3,*]
[1]UCL Institute of Ophthalmology
[2]UCL Institute of Neurology
[3]Department of Neuroscience, Physiology, and Pharmacology
University College London, London WC1E 6BT, UK
[4]Division of Biological Sciences, University of San Diego, San Diego, CA 92110, USA
[5]Werner Reichardt Centre for Integrative Neuroscience
[6]Graduate Training Centre of Neuroscience
University of Tübingen, 72074 Tübingen, Germany
[7]Present address: UCL Institute of Behavioural Neuroscience, Department of Experimental Psychology, 26 Bedford Way, London WC1H 0AP, UK
[8]Present address: Coulter Department of Biomedical Engineering, Georgia Institute of Technology and Emory University, Atlanta, GA 30332, USA
[9]Present address: Brain Research Institute, University of Zurich, 8057 Zürich, Switzerland
[10]Present address: Department of Biology II, Division of Neurobiology, LMU Munich, 80539 München, Germany
[11]Lead Contact
*Correspondence: aman.saleem@ucl.ac.uk (A.B.S.), m.carandini@ucl.ac.uk (M.C.), kenneth.harris@ucl.ac.uk (K.D.H.)
http://dx.doi.org/10.1016/j.neuron.2016.12.028

SUMMARY

Primary visual cortex exhibits two types of gamma rhythm: broadband activity in the 30–90 Hz range and a narrowband oscillation seen in mice at frequencies close to 60 Hz. We investigated the sources of the narrowband gamma oscillation, the factors modulating its strength, and its relationship to broadband gamma activity. Narrowband and broadband gamma power were uncorrelated. Increasing visual contrast had opposite effects on the two rhythms: it increased broadband activity, but suppressed the narrowband oscillation. The narrowband oscillation was strongest in layer 4 and was mediated primarily by excitatory currents entrained by the synchronous, rhythmic firing of neurons in the lateral geniculate nucleus (LGN). The power and peak frequency of the narrowband gamma oscillation increased with light intensity. Silencing the cortex optogenetically did not abolish the narrowband oscillation in either LGN firing or cortical excitatory currents, suggesting that this oscillation reflects unidirectional flow of signals from thalamus to cortex.

INTRODUCTION

Gamma rhythms are produced by a wide range of brain circuits and are thought to reflect multiple phenomena. Rhythms in a broad gamma range (30–90 Hz) have long been observed in regions including isocortex (Engel et al., 2001; Fries, 2009), hippocampus (Bragin et al., 1995), amygdala, striatum (Popescu et al., 2009), and cerebellum (Middleton et al., 2008). In cortex, these rhythms are believed to arise from the precisely timed synchronization of inhibitory networks (Cardin et al., 2009; Penttonen et al., 1998; Sohal et al., 2009; Wang and Buzsáki, 1996) and have been implicated in a wide range of functions including coherent transmission of information between neuronal assemblies (Fries, 2005, 2009), multiplexing of information (Lisman and Idiart, 1995), or binding of multiple features of a sensory scene (Singer, 1999). Recently, however, it has become clear that gamma rhythms reflect multiple phenomena, and that a single neuronal circuit can support multiple types of gamma rhythms. In hippocampal area CA1, for example, gamma rhythms occurring in distinct frequency bands are coherent with distinct input sources (Colgin et al., 2009), perhaps to provide separate routes for information from those structures. Understanding the origin of multiple patterns of high-frequency rhythms is essential for understanding the functional roles these rhythms might play.

In the visual cortex, there is evidence for two types of gamma rhythm, one with power distributed in a band between 30 and 90 Hz, and one that oscillates in a much narrower band. The gamma rhythms between 30 and 90 Hz have long been described in multiple species including cats (Eckhorn et al., 1988; Gray and Singer, 1989), primates (Fries et al., 2001; Kreiter and Singer, 1992; Pesaran et al., 2002), humans (Tallon et al., 1995), and mice (Cardin et al., 2009; Sohal et al., 2009; Welle and Contreras, 2016). We refer to these

© 2017 The Authors. Published by Elsevier Inc.
This is an open access article under the CC BY license (http://creativecommons.org/licenses/by/4.0/).

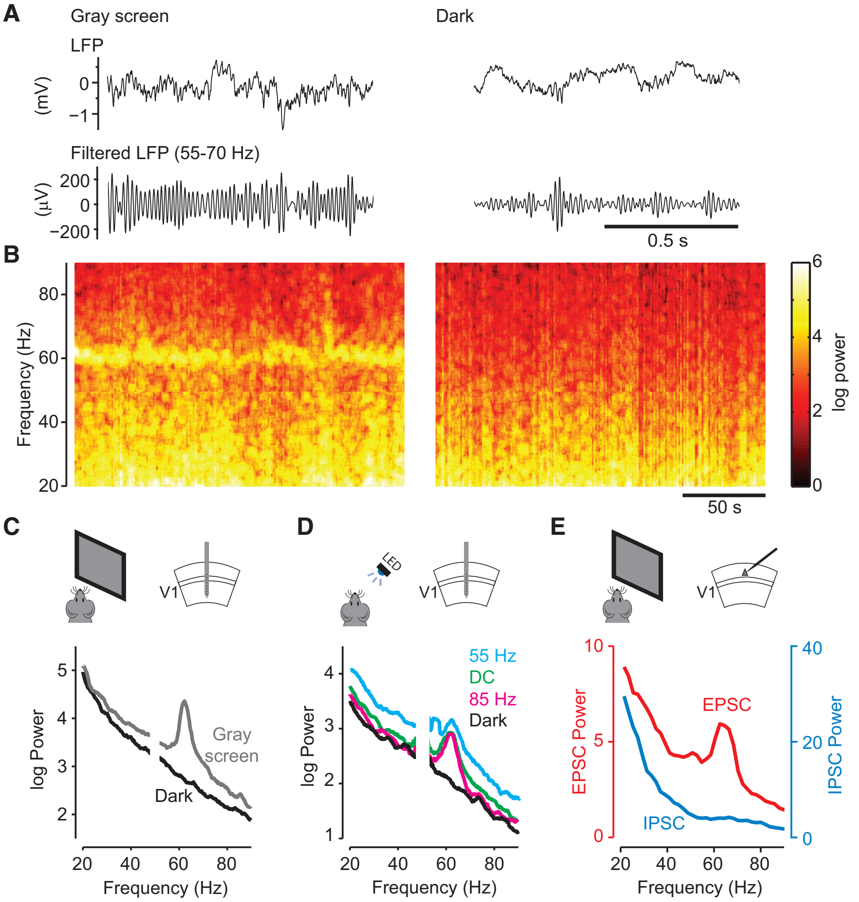

Figure 1. Narrowband Gamma in Mouse Visual Cortex

(A) Examples of local field potential (LFP) recorded in the primary visual cortex (V1) of mice running on a treadmill while viewing a uniform gray screen (left) or in complete darkness (right). Top traces: 1 s of single-trial broadband LFP; lower traces: same data, filtered between 55 and 70 Hz.

(B) Spectrograms showing the variation of LFP power over a longer timescale. The LFP was recorded in V1 of mice running on a treadmill during uniform gray (left) or complete darkness (right).

(C) Average LFP power spectra during uniform gray screen conditions (gray) or complete darkness (black) (same recording shown in B).

(D) LFP power spectra under various conditions of LED stimulation: flickering at 55 Hz (cyan), flickering at 85 Hz (magenta), constantly on (DC, green), and off (Dark, black) (example from one recording session).

(E) Power in the excitatory postsynaptic currents (EPSCs, red; whole-cell voltage-clamp recording at −70 mV) and inhibitory postsynaptic currents (IPSCs, blue; whole-cell voltage-clamp recording at +20 mV) in layer 2/3 neurons in V1, recorded during the uniform gray condition (average spectra across eleven recorded neurons).

rhythms between 30 and 90 Hz as broadband gamma. They are modulated by factors such as stimulus position, context, or cognitive state (Chalk et al., 2010; Fries et al., 2001; Gieselmann and Thiele, 2008; Gray and Singer, 1989; Pesaran et al., 2002). A fundamentally different gamma oscillation has been reported in visual cortex of anesthetized cats. This oscillation is extremely narrow in bandwidth in single experiments, and can appear also in the lateral geniculate nucleus (LGN) and retina (Koepsell et al., 2009, 2010; Neuenschwander and Singer, 1996). A gamma oscillation with a sharp band close to 60 Hz has also been observed in visual cortex of awake mice, where its power grows markedly with locomotion (Lee et al., 2014; Niell and Stryker, 2010). We refer to this oscillation as narrowband gamma, but note that some authors have used this term differently (Hermes et al., 2015a, 2015b; Ray and Maunsell, 2010, 2011; Scheeringa et al., 2011; Winawer et al., 2013). The origins of this oscillation, and the behavioral and sensory factors modulating it, are unknown.

Here we investigate the narrowband gamma oscillation in awake mouse primary visual cortex (V1) and establish the factors that determine its strength and frequency, and its relationship to broadband gamma activity. We find that this oscillation has different properties from broadband gamma activity: its amplitude increases with mean light intensity and with locomotion, but it decreases with visual contrast. The narrowband oscillation occurs independently of broadband gamma activity, indicating that it arises from different mechanisms. These mechanisms involve synaptic excitation more than inhibition and operate before the visual signals reach the cortex. Indeed, the narrowband oscillation is present earlier in the visual system, arising at least as early as the LGN.

RESULTS

We start by characterizing the narrowband gamma oscillation in mouse V1. We then explore its synaptic basis and its dependence on visual contrast and locomotion. Finally, we turn to the LGN and to optogenetic manipulations that investigate the source of the narrowband oscillation.

Narrowband Gamma in V1

Consistent with previous reports (Lee et al., 2014; Niell and Stryker, 2010), when we recorded in V1 of awake mice using multi-electrode arrays (Ayaz et al., 2013; Saleem et al., 2013) we observed a sharp peak in the local field potential (LFP) power spectrum close to 60 Hz (Figures 1A–1C and S1, available online). The frequency of this oscillation was 61 Hz in this example experiment (Figures 1B and 1C) and could vary between 55 and 70 Hz in other experiments, with a remarkably narrow bandwidth of 2–5 Hz. This oscillation could not have reflected mains interference, for these recordings were conducted in Europe, where (unlike in the United States) the electricity alternates at 50 Hz.

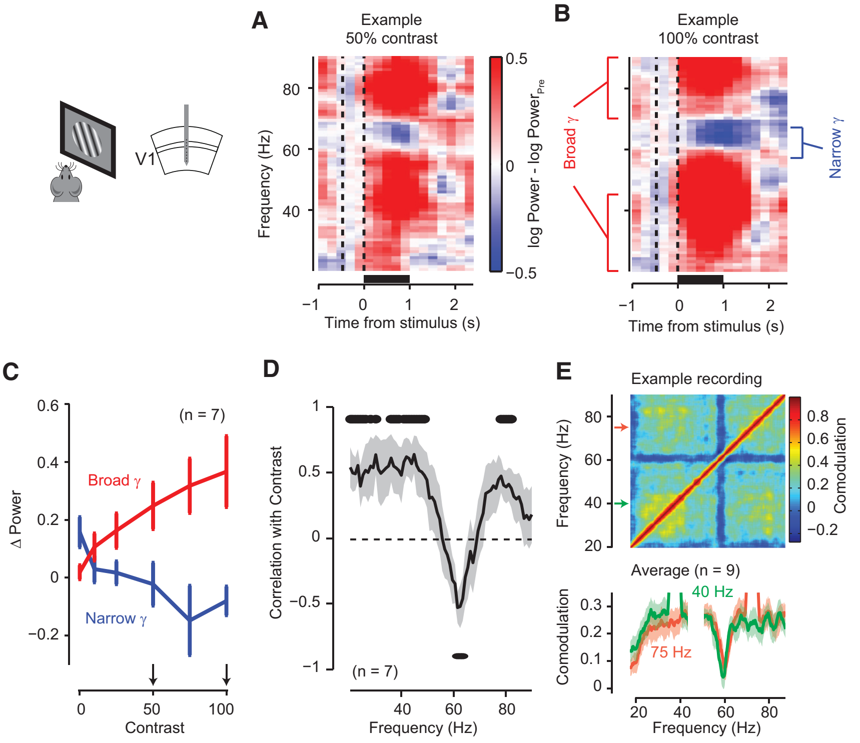

Figure 2. Narrowband Gamma Oscillations Are Suppressed by Visual Contrast

(A and B) LFP spectrogram averaged over 20 presentations of a drifting grating stimulus at 50% contrast (A) and 100% contrast (B) in a single experiment. Color shows power relative to pre-stimulus baseline (−0.5 to 0 s); bars below indicate the period of presentation of a 50° sinusoidal drifting grating in the retinotopic location of the recorded area.

(C) Power at the end of the stimulus presentation period (1 s) as a function of visual contrast for narrowband gamma (blue, calculated as the mean across the 55–65 Hz band) and broadband gamma (red, calculated as the mean across bands 20–40 and 70–90 Hz). The small increase in narrowband gamma power for zero contrast (blank) stimuli likely reflects continuing recovery from suppression by the preceding stimulus. Error bars show mean ± SEM.

(D) Correlation of power at different frequencies with contrast. Shaded area shows the mean ± SEM across experiments (n = 7); dots above/below the curve indicate frequencies with significant positive/negative correlations ($p < 0.05$; n = 7).

(E) Comodulation of power at different frequencies, i.e., the correlation in the power of different frequency bands, in an example recording. The blue cross visible at 60 Hz indicates that narrowband gamma power is uncorrelated with broadband gamma power. Bottom: comodulation of two frequencies of broadband gamma, 40 Hz (green) and 75 Hz (red), with respect to other frequency bands, showing a dip at narrowband gamma frequency. Shaded area shows the mean ± SEM across nine recordings in a virtual reality environment. Arrows indicate the frequencies shown in the bottom plots.

This narrowband oscillation was prominent when the mice viewed a uniform gray screen (50 cd/m^2), but it disappeared when we repeated the experiments in complete darkness (Figures 1A–1C; <10^{-2} cd/m^2). The oscillation was due to the overall light intensity, not the monitor refresh frequency (which can weakly entrain visual cortical neurons; Veit et al., 2011; Williams et al., 2004). We confirmed this with control experiments where we stimulated the visual field not with a monitor, but with a light-emitting diode (LED, 470 nm) generating light steadily with direct current (DC) input, or flickering at different rates. We saw a narrowband oscillation close to 60 Hz in all cases, independent of visual flicker rates (Figure 1D; $p > 0.1$, t test for all pairs of flicker rates; see Supplemental Experimental Procedures).

Unlike broadband gamma rhythms, which involve inhibitory currents (Cardin et al., 2009; Hasenstaub et al., 2005; Sohal et al., 2009; Welle and Contreras, 2016), the narrowband gamma oscillation was primarily mediated by excitatory synaptic currents (EPSCs) (Figure 1E). To uncover the synaptic basis of the narrowband oscillation, we analyzed intracellular recordings from layer 2/3 regular-spiking (putative pyramidal) neurons in V1 of awake mice viewing a uniform gray screen (Haider et al., 2013) (Figure 1E). EPSCs, isolated by voltage clamping the membrane potential near the reversal potential for inhibition, showed a clear peak around 60 Hz, similar to that seen in the extracellular LFP. The power in the narrowband gamma frequency range was significantly greater than a baseline prediction based on a smooth power spectrum that excludes this range (the "residual spectrum"; Figure S1; $p = 0.027$; t test over n = 11 recordings). However, we did not observe a similar peak in narrowband gamma power in the inhibitory synaptic currents (IPSCs) recorded from the same neurons during the same stimulus conditions ($p = 0.11$; n = 11 recordings). This suggests that at least in superficial layers, the narrowband gamma oscillation is primarily mediated by EPSCs.

Narrowband Gamma Is Suppressed by Visual Contrast

Another fundamental difference between narrowband and broadband gamma activity is that increasing visual contrast had opposite effects on them (Figures 2A–2D and S2). When we measured V1 responses in awake mice to drifting gratings (60° diameter, 2 cycles/s, 0.05 cycles/°) at different levels of visual contrast, we found a positive correlation of broadband power with contrast ($\rho = 0.59 \pm 0.18$; n = 7 recordings; Figures 2A–2D). For the narrowband gamma oscillation, instead, the power was instead negatively correlated with contrast ($\rho = -0.43 \pm 0.16$; n = 7 recordings; Figures 2C and 2D).

Narrowband gamma oscillation was suppressed by contrast not only during passive visual presentation, but also while mice used visual inputs to guide locomotion (Figure 2C). We recorded V1 activity while mice navigated in a virtual reality environment where visual cues indicated a position that mice must reach to receive a water reward. The narrowband gamma oscillation increased prominently during inter-trial periods, when the screen was uniform gray (Figure 2C; the increase in narrowband power

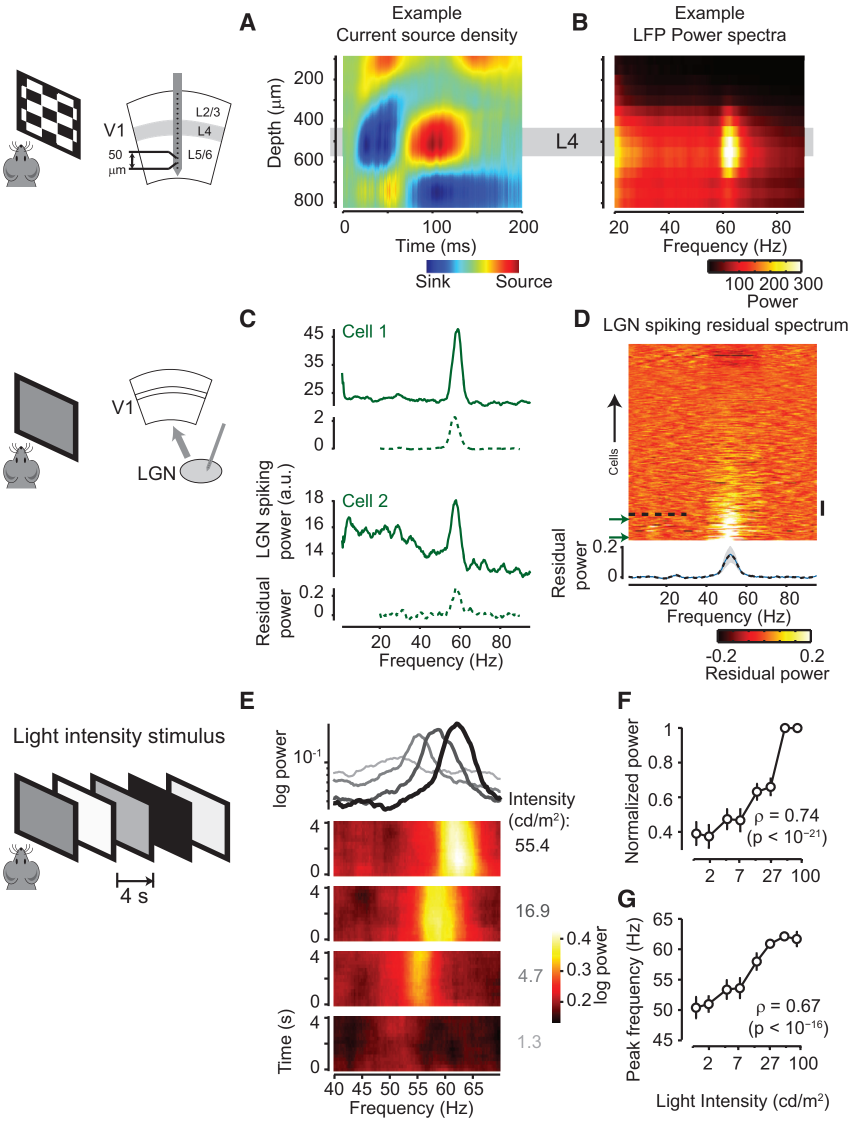

Figure 3. Narrowband Gamma Is Highest in L4 of V1 and Present in LGN Spiking Activity

(A) We recorded across the laminae of V1 using a 16-channel multi-electrode array. Current source density analysis was used to identify L4 as the location of the earliest current sink in response to a checkerboard stimulus (figure shows data from one representative experiment).

(B) LFP power spectrum as a function of depth in this same experiment, showing highest narrowband gamma power close to L4.

(C) Extracellular recordings from the LGN using a multi-electrode array. The spike train power spectra show a clear gamma modulation (two example neurons). Below each spectrum, the dotted line shows the fractional increase in power from a fit to the spectra excluding the narrowband gamma range (residual power spectrum).

(D) Pseudocolor plot showing residual spectra of all recorded LGN neurons (n = 323), ordered by narrowband gamma power. The mean ± SEM of the residual power across the population is shown at the bottom. Cells with residual gamma power over 0.20 are below the black dotted line (44 cells). The vertical scale bar indicates 20 cells, and the green arrows point to the examples shown in (C).

(E) We recorded activity in the LGN while presenting gray screen stimuli with light intensity changing every 4 s. From bottom: average spectrogram of spiking activity of a single neuron, triggered on the onset of four light intensities (1.3, 4.7, 16.9, and 55.4 cd/m^2; each intensity was presented 30 times). The mean power spectrum for each intensity is shown on the top (ranging from light gray for 1.3 to black for 55.4).

(F) Narrowband gamma power is positively correlated with light intensity ($\rho = 0.74$; $p < 10^{-21}$; n = 15 recordings). Error bars show mean ± SEM.

(G) The peak narrowband frequency is positively correlated with intensity ($\rho = 0.67$; $p < 10^{-16}$; n = 15 recordings).

prior to the offset of the previous trial likely reflects the uniform gray texture of the wall at the far end of the virtual corridor). Further, throughout the experiment the power of this narrowband gamma oscillation was uncorrelated with power in broadband gamma range (Figure 2E).

Narrowband Gamma in the LGN

The power of the narrowband gamma oscillation varied strongly across the depth of visual cortex and was markedly higher in layer 4 (L4; Figures 3A, 3B, and S3). We recorded the activity across different laminae of V1 using a linear multi-electrode array (16 sites, 50 µm spacing; Figure 3A) and identified L4 in each recording as the location showing the earliest current sink (Mitzdorf, 1985) in response to a contrast-reversing checkerboard stimulus (Figure 3A). This layer also showed the strongest ~60 Hz narrowband gamma peak (Figure 3B). The highest residual power was observed at the same depth as the largest current sink in all recordings (correlation $\rho = 0.98$; $p < 10^{-3}$; n = 6 recordings; Figure S3).

Because L4 receives strong inputs from the LGN, we next asked if the narrowband gamma oscillation was also present in these inputs, and we found that it is evident in many LGN neurons (Figures 3C and 3D). We used silicon probes to record from 323 LGN neurons from 7 awake head-fixed mice viewing a uniform gray screen (~50 cd/m^2). Inspection of spike-train power spectra revealed that many neurons fired rhythmically at frequencies close to 60 Hz (Figures 3C and 3D). We quantified the rhythmicity of each neuron as the power in the narrowband gamma frequency range compared to a baseline prediction based on a smooth power spectrum (the “residual spectrum”; Figure S1). This measure yielded high values in a sizeable proportion of LGN neurons (Figure 3D). For instance, 14% of LGN neurons (44/323; n = 18 recordings from 7 animals) showed a narrowband gamma peak greater than 20% of the baseline

prediction (dotted line in Figure 3D). Consistent with our results in V1 (Figure 2), the power of narrowband gamma in LGN decreased with increasing levels of contrast ($\rho = -0.53$; $p < 10^{-4}$; n = 9 recordings; Figures S4A–S4C).

The narrowband gamma oscillation entrained the activity of many LGN neurons in a coherent fashion (Figure S5). We observed the narrowband oscillation in the spike time autocorrelations (Figure S5A) and found that it was coherent between simultaneously recorded pairs of neurons (0.33 ± 0.04, mean ± SD; n = 215 cell pairs where both cells had over 20% residual power), being clearly visible in both the cross-correlogram and coherence spectrum computed from the spike trains of simultaneously recorded pairs (Figures S5B and S5C). Narrowband gamma in LGN therefore represents a network-wide oscillation, rather than independent oscillations in individual cells. Individual LGN neurons had diverse but consistent phases with respect to the oscillation: computing each neuron's preferred phase in two halves of the data yielded a similar estimate (correlation $\rho = 0.69$; $p < 10^{-6}$; Figure S5D). The strength and the phase of narrowband gamma entrainment in LGN neurons bore no obvious relationship to their visual preferences, such as receptive field polarity and contrast sensitivity (data not shown).

Narrowband Gamma Power and Frequency Increase with Light Intensity Levels

To investigate how narrowband gamma depends on light intensity, we recorded LGN neurons using extracellular multi-tetrode array recordings while varying the light intensity of a gray screen between ~1 and ~95 cd/m^2 (Figures 3E–3G). The light intensity changed every 4 s (Figure 3E), presented in interleaved sequences. The narrowband oscillation was seen when light intensities were above 4 cd/m^2, with power that increased with light intensity ($\rho = 0.74$; $p < 10^{-21}$; n = 15 recordings; Figures 3E, 3F, and S4D). Light intensity also had an effect on the oscillation's frequency, which increased from close to 50 Hz at low intensities (<5 cd/m^2) to around 60 Hz at high intensities (>30 cd/m^2). As a result, we found a strong correlation between the peak frequency of the narrowband gamma oscillation and the log light intensity level ($\rho = 0.67$; $p < 10^{-16}$; n = 15 recordings; Figure 3G).

Narrowband Gamma Is Increased in Active Animals

The strength of narrowband gamma oscillations was related not only to visual factors (light intensity and contrast) but also to the behavioral state of the animal. Consistent with previous reports (Lee et al., 2014; Niell and Stryker, 2010), we found that narrowband gamma power is highly correlated with running speed (correlation $\rho = 0.49 \pm 0.04$, mean ± SEM; n = 10 recordings in V1). This observation, however, does not imply that behavioral state itself directly modulates gamma power. Indeed, since the mouse pupil dilates during locomotion (Erisken et al., 2014; McGinley et al., 2015a, 2015b; Reimer et al., 2014; Vinck et al., 2015), and since narrowband gamma power depends on light intensity (Figure 3F), it remains possible that the correlation between locomotion and narrowband gamma reflects a greater amount of light striking the retina when pupils are dilated during running.

To answer this question, we measured gamma power after applying the anti-muscarinic agent tropicamide to the eye surface. This procedure caused full dilation of the pupil (Figure S6A), thus removing the correlation of pupil diameter with running speed. We found that the correlation of narrowband gamma power with running speed persisted even in these conditions of fixed pupil diameter (correlation $\rho = 0.35$; $p < 0.01$; Figure S6B), confirming that the correlation of narrowband gamma oscillation with locomotion does not simply reflect an indirect effect of increased light flux after pupil dilation.

Narrowband Gamma Does Not Require V1 Activity

The presence of the narrowband gamma oscillation in LGN suggests, but does not prove, that the cortex inherits this rhythm from the thalamus. Indeed, this oscillation could be generated in V1 independently of LGN activity. To investigate this question, we recorded activity in V1 using extracellular multi-electrode arrays, while silencing the activity in the LGN by optogenetically activating thalamic reticular nucleus (TRN) neurons (Reinhold et al., 2015). Silencing LGN strongly suppressed LFP activity in V1 at all frequencies, including the narrowband gamma peak close to 60 Hz (Figures S7A–S7C). This result indicates that the narrowband gamma oscillation cannot be sustained in V1 without input from the thalamus.

A second hypothesis is that the oscillation could rely on the presence of cortico-thalamic connections. To test this hypothesis, we asked whether silencing V1 would suppress the narrowband gamma oscillation in LGN and in the excitatory currents that it elicits in L4 cortical neurons. We performed these experiments in VGAT-ChR2 mice, where excitatory V1 activity could be silenced optogenetically across layers (Lien and Scanziani, 2013) (Figures S7D–S7I). To facilitate combined intracellular and extracellular recordings (Figure 4A), the mice were lightly anesthetized with urethane. This light anesthesia did not impede the narrowband gamma oscillation: LGN neurons showed rhythmic firing at frequencies close to 60 Hz, and EPSCs recorded simultaneously in L4 neurons showed a coherent oscillation (Figures 4B, 4D, and 4F).

Silencing the cortex optogenetically did not abolish the narrowband oscillation observed in LGN or the EPSCs in L4 neurons (Figures 4 and S7J–S7L). Silencing V1 caused no significant change in the frequency ($p = 0.312$; paired t test; n = 47 neurons with greater than 50% residual power in either condition; Figure 4B) and only a slight reduction of power ($p = 0.042$; Figure 4C) of the narrowband oscillation seen in LGN spike trains. While cortical silencing reduced L4 EPSC spectral power at all frequencies, there was actually a slight increase in the residual power of the narrowband gamma oscillation ($p = 0.047$; n = 14; Figures 4D, 4E, and S7L), consistent with addition of a narrowband oscillation inherited from the thalamus, to cortically generated broadband activity. The cross-coherence between LGN spiking activity and the EPSCs in L4 V1 neurons showed a clear peak at the narrowband gamma frequencies, which was unaffected by cortical silencing ($p = 0.15$; n = 47; Figures 4F and 4G). These results demonstrate that the narrowband gamma oscillation in LGN persists in the absence of feedback from V1.

DISCUSSION

We have shown that the narrowband gamma oscillation in mouse V1 has several major differences from the more

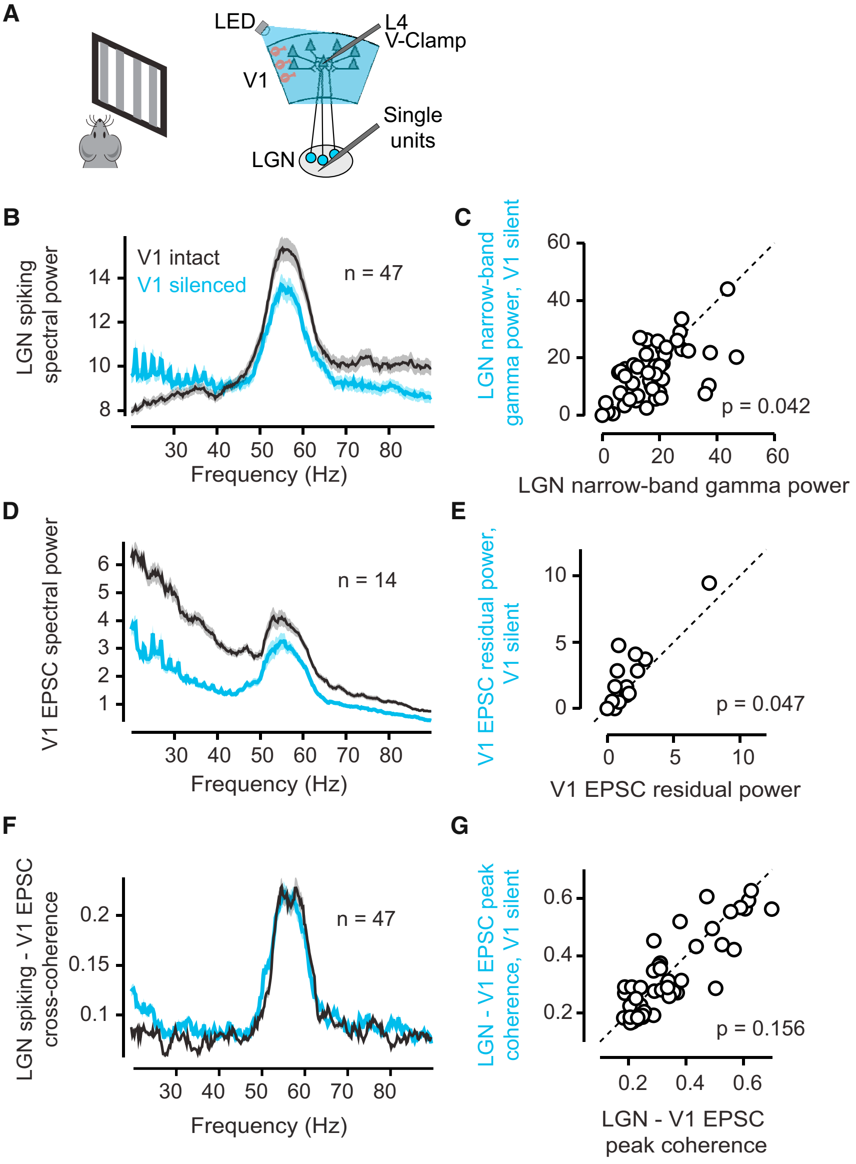

Figure 4. Narrowband Gamma Oscillations in LGN and V1 EPSCs Persist in the Absence of V1 Spiking

(A) We made whole-cell voltage-clamp recordings of EPSCs in neurons in L4 of V1 while simultaneously recording single-unit activity in the LGN of VGAT-ChR2 mice under light urethane anesthesia. V1 could be silenced by shining a blue LED over the region.

(B) Mean (±SEM) spectral power across LGN spike trains (n = 47 neurons) when V1 activity was intact (black) or silenced (cyan).

(C) Comparison of the peak power of the narrowband gamma when cortical activity is intact or silenced (n = 47 neurons).

(D) Mean (±SEM) EPSC spectral power across neurons with and without cortical silencing (n = 14 neurons).

(E) Comparison of residual narrowband gamma power when cortical activity is intact or silenced (n = 14 neurons).

(F) The cross-coherence between EPSCs recorded in V1 and LGN spiking activity shows a peak at the narrowband gamma frequency, which does not change with cortical silencing (n = 47 pairs).

(G) Comparison of maximum LGN-V1 cross-coherence when cortical activity is intact or silenced (n = 47 neurons).

commonly described broadband gamma rhythm. The power of narrow- and broadband gamma activity was uncorrelated, and visual contrast suppressed the narrowband gamma oscillation while it increased broadband gamma rhythms. Within cortex, the narrowband gamma oscillation was strongest in L4, and cortical intracellular recordings revealed narrowband gamma in excitatory, but not inhibitory, synaptic currents. Narrowband gamma oscillations persisted in LGN ensembles and L4 synaptic inputs, even after silencing of cortex.

We therefore conclude that the narrowband gamma oscillation (2–5 Hz bandwidth oscillation close to 60 Hz) is generated in thalamus and possibly even retina, representing an entirely different phenomenon than broadband gamma (30–90 Hz rhythms). Whereas broadband gamma rhythms involve cortical inhibitory networks (Cardin et al., 2009; Penttonen et al., 1998; Sohal et al., 2009; Wang and Buzsáki, 1996), narrowband gamma is transmitted to cortex by the rhythmic firing of thalamic ensembles. Although resonance in thalamocortical loops has been proposed as a mechanism for the generation of high-frequency oscillations (Steriade, 1997, 2000), we found that the narrowband gamma oscillation was transmitted from LGN to visual cortex even after cortical firing was abolished optogenetically. We therefore propose that the oscillation is generated either within thalamic circuits or by their interactions with their retinal inputs.

Our results are consistent with previous reports of narrowband oscillations in mouse visual cortex at frequencies close to 60 Hz (Lee et al., 2014; Niell and Stryker, 2010). Furthermore, the oscillation we study here might be homologous to an oscillation previously described in LGN of anesthetized cats (Castelo-Branco et al., 1998; Koepsell et al., 2009, 2010; Neuenschwander and Singer, 1996), which showed extremely narrowband rhythmicity within single recordings. The frequency of that oscillation, however, varied between recordings, spanning a range of 45–114 Hz. In these cat recordings, the thalamic oscillation was highly synchronous with a retinal oscillation determined either by simultaneous recording or by analysis of intracellular excitatory postsynaptic potentials (EPSPs) in thalamus. If this proposed homology is correct, it would suggest that the narrowband gamma recorded here might reflect an oscillation generated in retina and transmitted through LGN to visual cortex. The fact that the power of this oscillation is increased by locomotion (as well as by stimulation of mesencephalic locomotor nuclei

at levels too small to evoke locomotion; Lee et al., 2014) suggests that its amplitude is likely to be modulated by thalamic circuitry.

What function might this narrowband gamma oscillation play in visual processing? Although a definitive answer to this question requires further work, the present data are sufficient to formulate two hypotheses.

A first hypothesis is that the narrowband gamma oscillation represents an ''idling'' rhythm. In this interpretation, narrowband gamma represents a default state of LGN activity when visual processing is not being carried out, similar to the proposed function of the visual alpha rhythm and the beta oscillation of the motor system. This hypothesis would explain why this oscillation is suppressed by increases in visual contrast.

In the second hypothesis, the narrow- and broadband gamma oscillations would represent specific channels for thalamocortical and corticocortical communication. It has been proposed that oscillatory patterns with different frequency characteristics may allow routing of information between specific brain structures. For example, the coherence of slow gamma between hippocampal areas CA1 and CA3, and fast gamma between CA1 and entorhinal cortex (Colgin et al., 2009), may indicate the existence of two separate transmission channels in the hippocampal formation. Analogously, the narrowband gamma oscillation may provide a specific channel for feedforward transmission from LGN to visual cortex, while broadband gamma—which is generated in cortical regions—enables transmission of information between cortical circuits.

EXPERIMENTAL PROCEDURES

Please see the Supplemental Experimental Procedures for details of all methods.

SUPPLEMENTAL INFORMATION

Supplemental Information includes Supplemental Experimental Procedures, seven figures, and one table and can be found with this article online at http://dx.doi.org/10.1016/j.neuron.2016.12.028.

AUTHOR CONTRIBUTIONS

Conceptualization, A.B.S., M.K., M.C., and K.D.H.; Methodology, A.B.S., A.D.L., B.H., M.R.R., A.A., K.R., and L.B. (specific experimental contributions are listed in Table S1); Formal Analysis, A.B.S.; Writing – Original Draft, A.B.S., M.C., and K.D.H.; Writing – Review & Editing, all authors. M.C. and K.D.H. are co-senior authors.

ACKNOWLEDGMENTS

A.B.S., M.C., and K.D.H. are funded by the Wellcome Trust (grants 095668 and 095669) and by the Simons Collaboration in the Global Brain (grant 325512). K.D.H. is also funded by the EPSRC (K015141). M.R.R. and L.B. were supported by funds awarded to the Centre for Integrative Neuroscience (DFG EXC 307). A.D.L. and K.R. performed the experiments in Massimo Scanziani's lab with the support of the Howard Hughes Medical Institute and the Gatsby Charitable Foundation. K.R. was also supported by the National Science Foundation Graduate Research Program Fellowship. We thank R. Dulinskas for help with data collection for Figure S3. M.C. holds the GlaxoSmithKilne/Fight for Sight Chair in Visual Neuroscience.

Received: April 15, 2016
Revised: October 4, 2016
Accepted: December 2, 2016
Published: January 18, 2017

REFERENCES

Ayaz, A., Saleem, A.B., Schölvinck, M.L., and Carandini, M. (2013). Locomotion controls spatial integration in mouse visual cortex. Curr. Biol. *23*, 890–894.

Bragin, A., Jandó, G., Nádasdy, Z., Hetke, J., Wise, K., and Buzsáki, G. (1995). Gamma (40–100 Hz) oscillation in the hippocampus of the behaving rat. J. Neurosci. *15*, 47–60.

Cardin, J.A., Carlén, M., Meletis, K., Knoblich, U., Zhang, F., Deisseroth, K., Tsai, L.H., and Moore, C.I. (2009). Driving fast-spiking cells induces gamma rhythm and controls sensory responses. Nature *459*, 663–667.

Castelo-Branco, M., Neuenschwander, S., and Singer, W. (1998). Synchronization of visual responses between the cortex, lateral geniculate nucleus, and retina in the anesthetized cat. J. Neurosci. *18*, 6395–6410.

Chalk, M., Herrero, J.L., Gieselmann, M.A., Delicato, L.S., Gotthardt, S., and Thiele, A. (2010). Attention reduces stimulus-driven gamma frequency oscillations and spike field coherence in V1. Neuron *66*, 114–125.

Colgin, L.L., Denninger, T., Fyhn, M., Hafting, T., Bonnevie, T., Jensen, O., Moser, M.B., and Moser, E.I. (2009). Frequency of gamma oscillations routes flow of information in the hippocampus. Nature *462*, 353–357.

Eckhorn, R., Bauer, R., Jordan, W., Brosch, M., Kruse, W., Munk, M., and Reitboeck, H.J. (1988). Coherent oscillations: a mechanism of feature linking in the visual cortex? Multiple electrode and correlation analyses in the cat. Biol. Cybern. *60*, 121–130.

Engel, A.K., Fries, P., and Singer, W. (2001). Dynamic predictions: oscillations and synchrony in top-down processing. Nat. Rev. Neurosci. *2*, 704–716.

Erisken, S., Vaiceliunaite, A., Jurjut, O., Fiorini, M., Katzner, S., and Busse, L. (2014). Effects of locomotion extend throughout the mouse early visual system. Curr. Biol. *24*, 2899–2907.

Fries, P. (2005). A mechanism for cognitive dynamics: neuronal communication through neuronal coherence. Trends Cogn. Sci. *9*, 474–480.

Fries, P. (2009). Neuronal gamma-band synchronization as a fundamental process in cortical computation. Annu. Rev. Neurosci. *32*, 209–224.

Fries, P., Reynolds, J.H., Rorie, A.E., and Desimone, R. (2001). Modulation of oscillatory neuronal synchronization by selective visual attention. Science *291*, 1560–1563.

Gieselmann, M.A., and Thiele, A. (2008). Comparison of spatial integration and surround suppression characteristics in spiking activity and the local field potential in macaque V1. Eur. J. Neurosci. *28*, 447–459.

Gray, C.M., and Singer, W. (1989). Stimulus-specific neuronal oscillations in orientation columns of cat visual cortex. Proc. Natl. Acad. Sci. USA *86*, 1698–1702.

Haider, B., Häusser, M., and Carandini, M. (2013). Inhibition dominates sensory responses in the awake cortex. Nature *493*, 97–100.

Hasenstaub, A., Shu, Y., Haider, B., Kraushaar, U., Duque, A., and McCormick, D.A. (2005). Inhibitory postsynaptic potentials carry synchronized frequency information in active cortical networks. Neuron *47*, 423–435.

Hermes, D., Miller, K.J., Wandell, B.A., and Winawer, J. (2015a). Gamma oscillations in visual cortex: the stimulus matters. Trends Cogn. Sci. *19*, 57–58.

Hermes, D., Miller, K.J., Wandell, B.A., and Winawer, J. (2015b). Stimulus dependence of gamma oscillations in human visual cortex. Cereb. Cortex *25*, 2951–2959.

Koepsell, K., Wang, X., Vaingankar, V., Wei, Y., Wang, Q., Rathbun, D.L., Usrey, W.M., Hirsch, J.A., and Sommer, F.T. (2009). Retinal oscillations carry visual information to cortex. Front. Syst. Neurosci. *3*, 4.

Koepsell, K., Wang, X., Hirsch, J.A., and Sommer, F.T. (2010). Exploring the function of neural oscillations in early sensory systems. Front. Neurosci. *4*, 53.

Kreiter, A.K., and Singer, W. (1992). Oscillatory neuronal responses in the visual cortex of the awake macaque monkey. Eur. J. Neurosci. *4*, 369–375.

Lee, A.M., Hoy, J.L., Bonci, A., Wilbrecht, L., Stryker, M.P., and Niell, C.M. (2014). Identification of a brainstem circuit regulating visual cortical state in parallel with locomotion. Neuron *83*, 455–466.

Lien, A.D., and Scanziani, M. (2013). Tuned thalamic excitation is amplified by visual cortical circuits. Nat. Neurosci. *16*, 1315–1323.

Lisman, J.E., and Idiart, M.A. (1995). Storage of 7 ± 2 short-term memories in oscillatory subcycles. Science *267*, 1512–1515.

McGinley, M.J., David, S.V., and McCormick, D.A. (2015a). Cortical membrane potential signature of optimal states for sensory signal detection. Neuron *87*, 179–192.

McGinley, M.J., Vinck, M., Reimer, J., Batista-Brito, R., Zagha, E., Cadwell, C.R., Tolias, A.S., Cardin, J.A., and McCormick, D.A. (2015b). Waking state: rapid variations modulate neural and behavioral responses. Neuron *87*, 1143–1161.

Middleton, S.J., Racca, C., Cunningham, M.O., Traub, R.D., Monyer, H., Knöpfel, T., Schofield, I.S., Jenkins, A., and Whittington, M.A. (2008). High-frequency network oscillations in cerebellar cortex. Neuron *58*, 763–774.

Mitzdorf, U. (1985). Current source-density method and application in cat cerebral cortex: investigation of evoked potentials and EEG phenomena. Physiol. Rev. *65*, 37–100.

Neuenschwander, S., and Singer, W. (1996). Long-range synchronization of oscillatory light responses in the cat retina and lateral geniculate nucleus. Nature *379*, 728–732.

Niell, C.M., and Stryker, M.P. (2010). Modulation of visual responses by behavioral state in mouse visual cortex. Neuron *65*, 472–479.

Penttonen, M., Kamondi, A., Acsády, L., and Buzsáki, G. (1998). Gamma frequency oscillation in the hippocampus of the rat: intracellular analysis in vivo. Eur. J. Neurosci. *10*, 718–728.

Pesaran, B., Pezaris, J.S., Sahani, M., Mitra, P.P., and Andersen, R.A. (2002). Temporal structure in neuronal activity during working memory in macaque parietal cortex. Nat. Neurosci. *5*, 805–811.

Popescu, A.T., Popa, D., and Paré, D. (2009). Coherent gamma oscillations couple the amygdala and striatum during learning. Nat. Neurosci. *12*, 801–807.

Ray, S., and Maunsell, J.H. (2010). Differences in gamma frequencies across visual cortex restrict their possible use in computation. Neuron *67*, 885–896.

Ray, S., and Maunsell, J.H. (2011). Different origins of gamma rhythm and high-gamma activity in macaque visual cortex. PLoS Biol. *9*, e1000610.

Reimer, J., Froudarakis, E., Cadwell, C.R., Yatsenko, D., Denfield, G.H., and Tolias, A.S. (2014). Pupil fluctuations track fast switching of cortical states during quiet wakefulness. Neuron *84*, 355–362.

Reinhold, K., Lien, A.D., and Scanziani, M. (2015). Distinct recurrent versus afferent dynamics in cortical visual processing. Nat. Neurosci. *18*, 1789–1797.

Saleem, A.B., Ayaz, A., Jeffery, K.J., Harris, K.D., and Carandini, M. (2013). Integration of visual motion and locomotion in mouse visual cortex. Nat. Neurosci. *16*, 1864–1869.

Scheeringa, R., Fries, P., Petersson, K.M., Oostenveld, R., Grothe, I., Norris, D.G., Hagoort, P., and Bastiaansen, M.C. (2011). Neuronal dynamics underlying high- and low-frequency EEG oscillations contribute independently to the human BOLD signal. Neuron *69*, 572–583.

Singer, W. (1999). Neuronal synchrony: a versatile code for the definition of relations? Neuron *24*, 49–65, 111–125.

Sohal, V.S., Zhang, F., Yizhar, O., and Deisseroth, K. (2009). Parvalbumin neurons and gamma rhythms enhance cortical circuit performance. Nature *459*, 698–702.

Steriade, M. (1997). Synchronized activities of coupled oscillators in the cerebral cortex and thalamus at different levels of vigilance. Cereb. Cortex *7*, 583–604.

Steriade, M. (2000). Corticothalamic resonance, states of vigilance and mentation. Neuroscience *101*, 243–276.

Tallon, C., Bertrand, O., Bouchet, P., and Pernier, J. (1995). Gamma-range activity evoked by coherent visual stimuli in humans. Eur. J. Neurosci. *7*, 1285–1291.

Veit, J., Bhattacharyya, A., Kretz, R., and Rainer, G. (2011). Neural response dynamics of spiking and local field potential activity depend on CRT monitor refresh rate in the tree shrew primary visual cortex. J. Neurophysiol. *106*, 2303–2313.

Vinck, M., Batista-Brito, R., Knoblich, U., and Cardin, J.A. (2015). Arousal and locomotion make distinct contributions to cortical activity patterns and visual encoding. Neuron *86*, 740–754.

Wang, X.J., and Buzsáki, G. (1996). Gamma oscillation by synaptic inhibition in a hippocampal interneuronal network model. J. Neurosci. *16*, 6402–6413.

Welle, C.G., and Contreras, D. (2016). Sensory-driven and spontaneous gamma oscillations engage distinct cortical circuitry. J. Neurophysiol. *115*, 1821–1835.

Williams, P.E., Mechler, F., Gordon, J., Shapley, R., and Hawken, M.J. (2004). Entrainment to video displays in primary visual cortex of macaque and humans. J. Neurosci. *24*, 8278–8288.

Winawer, J., Kay, K.N., Foster, B.L., Rauschecker, A.M., Parvizi, J., and Wandell, B.A. (2013). Asynchronous broadband signals are the principal source of the BOLD response in human visual cortex. Curr. Biol. *23*, 1145–1153.

Neuron, Volume *93*

Supplemental Information

Subcortical Source and Modulation of the Narrowband Gamma Oscillation in Mouse Visual Cortex

Aman B. Saleem, Anthony D. Lien, Michael Krumin, Bilal Haider, Miroslav Román Rosón, Asli Ayaz, Kimberly Reinhold, Laura Busse, Matteo Carandini, and Kenneth D. Harris

Supplementary Figures

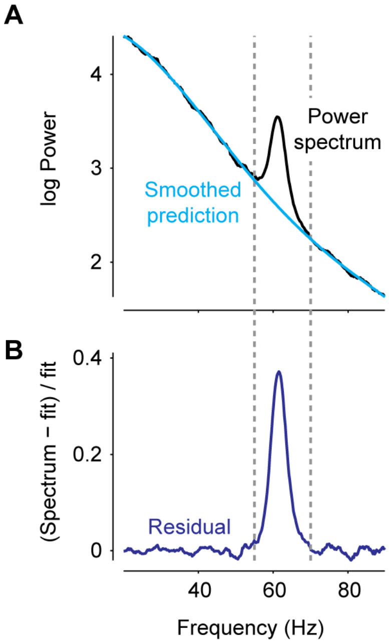

Figure S1 (related to Figure 1): Analysis of narrow-band oscillation. A. Example power spectrum from one recording session (Black). The power spectrum (excluding the narrow-band gamma region highlighted by the dotted lines) is fitted by a 4th order polynomial (smoothed prediction, cyan). **B.** The residual spectrum is the difference between the actual data and the fitted function, divided by the fitted function. The peak frequency of narrow-band gamma was calculated as the position of the peak in the residual spectrum.

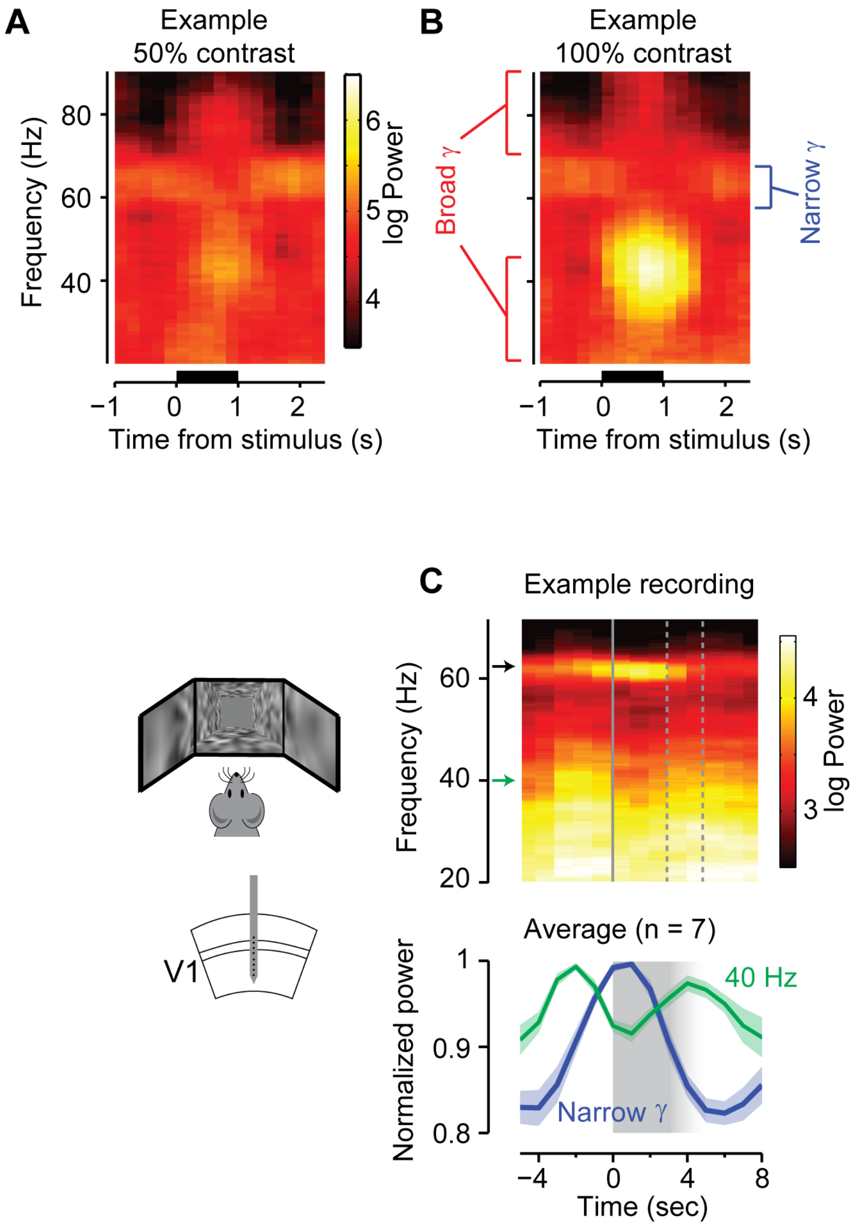

Figure S2 (related to Figure 2): Broadband gamma rhythms are enhanced by visual contrast while the narrowband gamma oscillation is suppressed. A-B. Average LFP spectrogram triggered on presentation of a drifting grating stimulus at 50% contrast (**A**) and 100% contrast (**B**), in a single experiment. Bars below indicate the period of presentation of a 50° sinusoidal drifting grating in the retinotopic location of the recorded area. **C.** Average LFP spectrogram triggered on the onset of a gray screen period in a virtual reality environment. The gray screen period lasted between 3-5 secs after which the animal re-enters the high contrast virtual environment. **(Bottom)** normalized power (power / maximal power) at the narrow band frequency (Narrowband gamma; Blue) and at 40 Hz (Green). The shaded regions show the mean ± SEM across 7 recording sessions. The gray shaded region shows the period of gray screen presentation, which varied between 2 to 4 s. Arrows in **C** indicate the frequencies shown in the bottom plots.

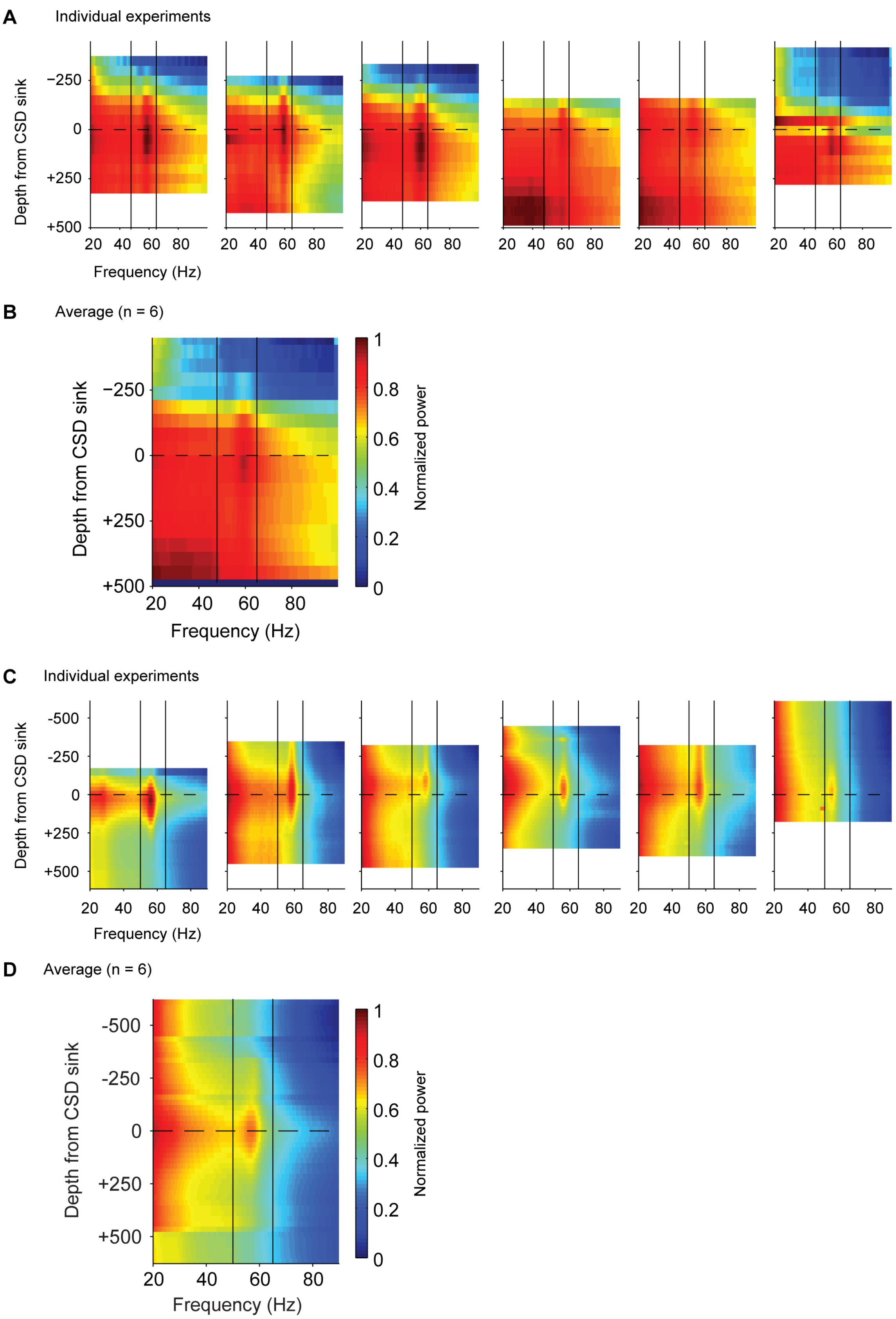

Figure S3 (related to Figure 3): Narrowband gamma is highest in Layer 4 of V1 across experiments. A,C. The power across frequencies as a function of depth from the putative Layer 4 (peak of the CSD sink) across six experiments. The vertical lines mark the frequency range (50-65Hz) of narrow-band

gamma oscillation. **B, D.** The depth profile of narrow band gamma averaged across all experiments. Experiments in A-B were conducted using protocol A and experiments in C-D were conducted using protocol B. We thank R. Dulinskas for help with data collection (C, D).

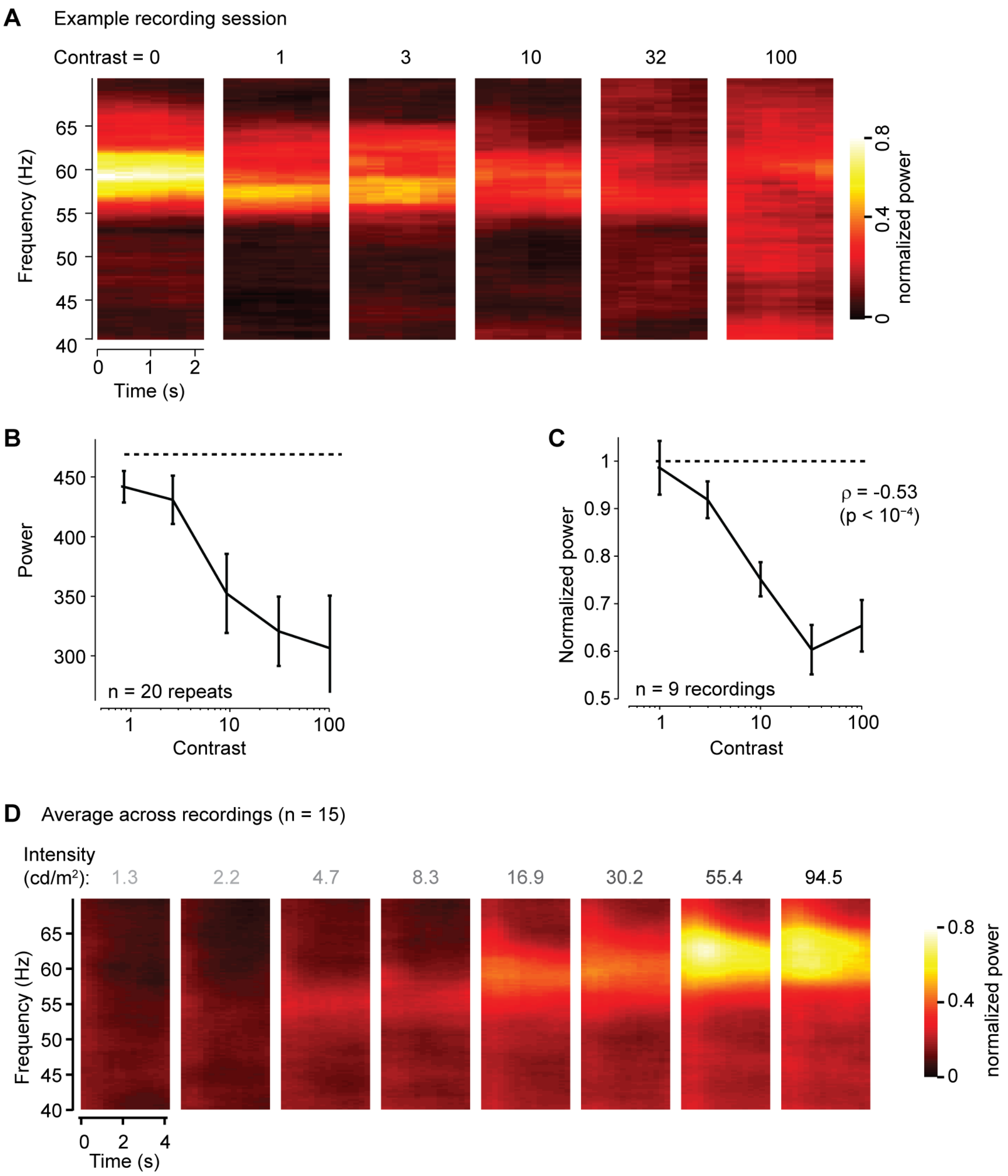

Figure S4 (related to Figures 2 & 3): Narrowband gamma oscillation in LGN spiking is suppressed by contrast. A. Average spectrogram of spiking activity of a single neuron, triggered on the start of full-screen grating stimuli (0%, 1%, 3%, 10%, 32% & 100% contrast, each contrast level repeated 20 times). The stimulus was presented for 2s, with a 3s inter-stimulus interval with 0% contrast (iso-luminant gray screen). **B.** Narrowband power decreases as a function of the contrast presented for the recording session shown in **A**. Errorbars show mean ± SEM. The dotted line shows the mean narrowband power at 0% contrast. **C.** Same as B, averaged across all the recording sessions (ρ = -0.53, $p<10^{-4}$; n = 9 recordings). The peak narrowband power was normalized for each recording based on the power at 0% contrast. **D.** Average spectrogram of spiking activity across all recording sessions for the 8 different intensities presented (similar to Figure 3E), showing an increase in gamma power with intensity (n = 15 recordings). The spectrograms of each recording session were normalized by the maximum average power in each experiment, before averaging across recordings.

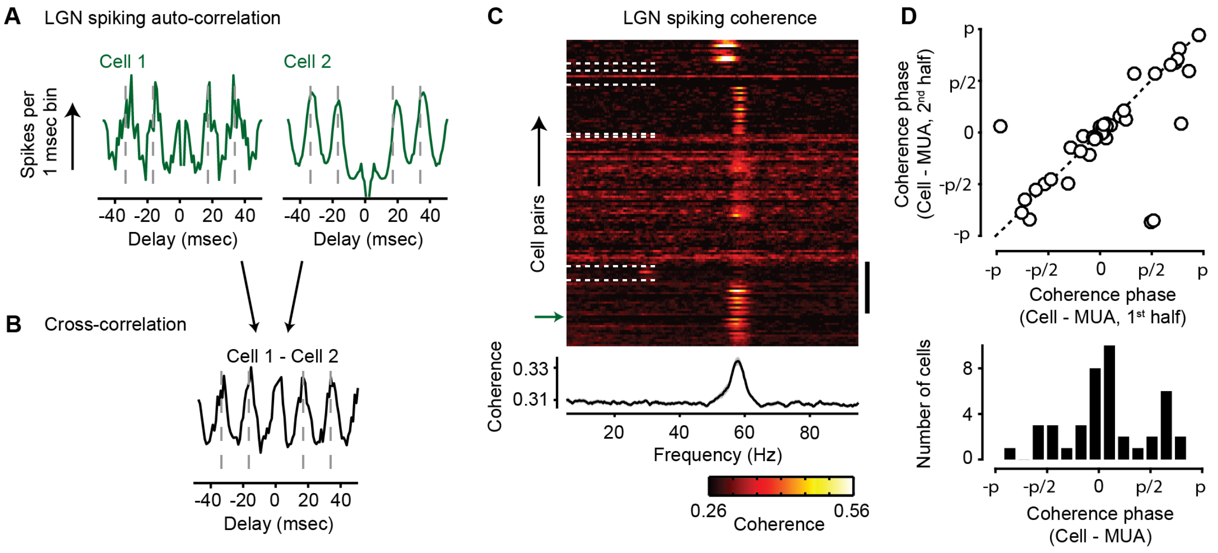

Figure S5 (related to Figure 3): Narrowband gamma is coherent between LGN units. A. Two examples of the spike auto-correlation of LGN neurons (same cells as C), showing a clear oscillation at narrowband gamma frequency. Dashed lines indicate the positions of the peaks expected for a 60 Hz modulation (at ±16.7 and ±33.4 msec). **B.** The cross-correlation of spike times of neurons in G. **C.** Coherence spectra for all pairs of simultaneously recorded neurons with residual gamma power greater than 0.20 (n = 130 pairs). White dotted lines separate pairs from different recording sessions. The mean ± SEM of the coherence spectra across all cell pairs is shown at the bottom. The scale bar indicates 20 cell pairs, and the green arrow points to the example cell pair shown in panels F. **D.** (Top) Preferred phase of each oscillatory cell relative to the population of other simultaneously recorded neurons, calculated in the first and second halves of the recording. (Bottom) The distribution of the preferred phase across cells (n = 44 cells).

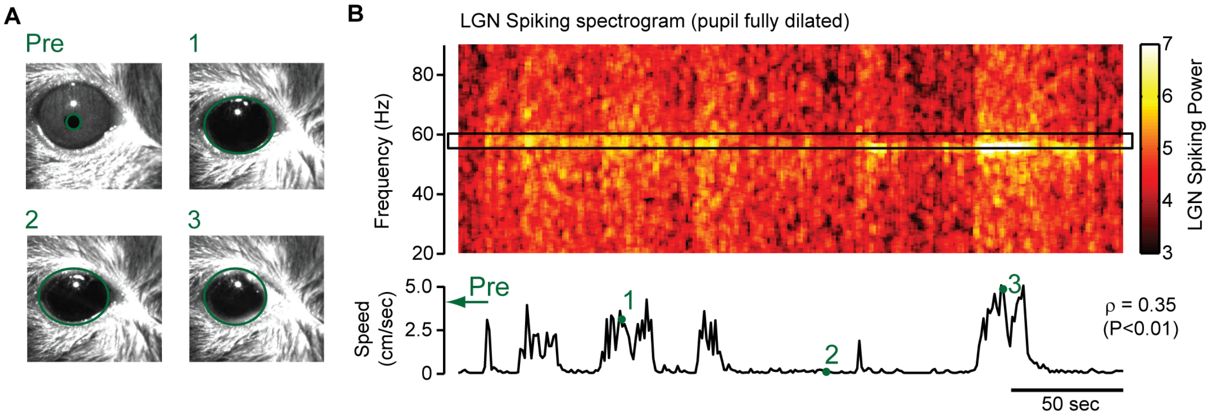

Figure S6 (related to Figure 3): Narrowband gamma power is correlated with running even with a fully dilated pupil. A. Images of the eye before dilation (Pre), and after being dilated (Image 1-3) with Tropicamide (>10 mins after full dilation), when the animal was stationary (Image 2) or running (Images 1 and 3). The green ovals over the images indicate the detected pupil area. **B.** Spectrogram of LGN spiking activity at different frequencies as a function of time. The trace below shows the running speed of the animal. Narrowband gamma power (mean in region highlighted by box) was positively correlated with running speed ($\rho = 0.35$, $p<0.01$). The time points of the images are shown in **A** are marked with green numerals.

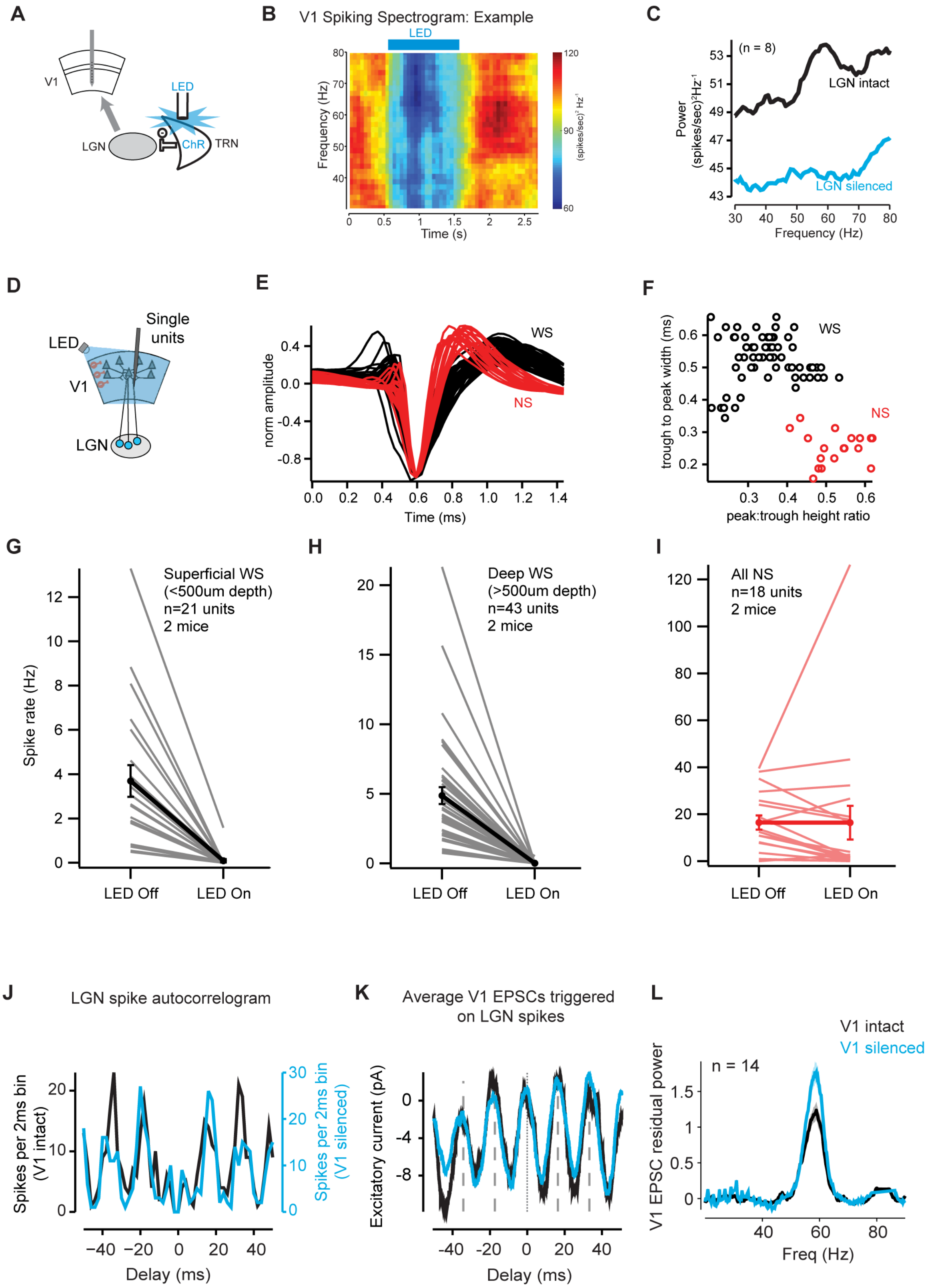

Figure S7 (related to Figure 4): Narrowband gamma oscillation in V1 is abolished by LGN inactivation, but not by V1 inactivation. A. We recorded activity in V1 using a multi-electrode array (in awake mice), while inactivating the LGN by activating the TRN using an LED. **B.** Spiking spectrogram of V1 activity triggered on LED stimulation (Example experiment) **C.** The average power of V1 during (cyan) and after (black) LGN inactivation (n = 8 experiments). The narrowband gamma

peak is abolished, together with a decrease in power at all frequencies. **D.** We recorded single-unit activity in the V1 of VGAT-ChR2 mice under light urethane anesthesia, where V1 could be silenced by shining a blue LED over the region. **E.** The plot shows superimposed waveforms of V1 neurons, color coded based on whether they were classified as wide spiking (WS, black) or narrow spiking (NS, red). **F.** The neurons were classified as WS or NS based on the peak-trough height ratio and trough to peak width (based on methods from Niell & Stryker, 2008). **G.** Turning on the LED suppressed the firing of all the WS neurons recorded from superficial layers (<500um; n = 21 neurons). **H.** Turning on the LED suppressed the firing of all the WS neurons recorded from deep layers (>500um; n = 43 neurons). **I.** The activity of NS neurons did not change significantly between the LED On and LED Off conditions (all depths; n = 18 neurons). **J.** Spike time autocorrelation of an LGN single unit when V1 activity was intact (black) or silenced (cyan) **K.** Average EPSCs recorded by voltage-clamping a V1 layer 4 neuron at -70mV, triggered on spikes of an LGN neuron (same neuron as in B, D), when V1 activity was intact (black) or silenced (cyan). Excitatory current negative. **L.** Mean (± SEM) residual power of the EPSC spectral power across neurons without and without cortical silencing (n = 14 neurons; related to Figure 4D).

Table S1 (related to Figure 1-4): Recording list. List of animal strains, recording electrodes, stimuli and data analyses parameters

Figure number	Animal strain (number of recordings)	Sex and number of animals	Electrodes	Stimuli	Parameters for analysis (Chronux)	Experimenter (Protocol followed)
1A,D	PV-Cre x Ai32 (2 recordings, 1 example shown)	2M	Neuronexus A2x4 tet	Flickering LED	Tapers: 5,9 Window: 3s, 1s shift	ABS (A)
1B,C	C57BL6 (1 example recording)	1M	Neuronexus A1x16	Gray screen / Dark	Tapers: 5,9 Window: 3s, 1s shift	ABS (A)
1E	C57BL6 (11 cells)	9F	Glass electrodes	Gray screen	Tapers: 3,2 Window: 0.33s, 0.5s shift	BH (A)
2A-D	C57BL6 (7 recordings)	3M	Neuronexus A1x16	Contrast, grating (2cycles/s, 0.05cycles/^{0})	Tapers: 5,9 Window: 1s, 0.2s shift	AA & ABS (A)
2E	C57BL6 (9 recordings)	3M	Neuronexus A1x32Edge or A2x4 tet	Virtual reality	Tapers: 5,9 Window: 3s, 1s shift	ABS (A)
3A-B	C57BL6 (6 recordings)	3M	Neuronexus A1x16	Contrast reversing checkerboard (3A; 15^0 squares, 2 cycles/s) or gray screen (3B)	Tapers: 5,9 Window: 3s, 1s shift	ABS & AA (A)
3C-D	C57BL6 (18 recordings)	2M / 5F	Neuronexus A1x32Edge	Gray screen	Tapers: 5,9 Window: 3s, 1s shift	MRR (B)
3E-F	C57BL6 (15 recordings)	3F	Neuronexus A2x4 tet	Full-field intensity & contrast	Tapers: 5,9 Window: 3s, 1s shift	ABS (A)
4	VGAT-ChR2 (14 whole-cell & 12 LGN recordings)	12 (M/F)	Glass electrodes + Neuronexus Buzsaki32	Full-field moving grating	Tapers: 3,5 Window: 2.5s	TL (C)
S1	C57BL6 (1 recording)	1M	Neuronexus A2x4 tet	Virtual reality	Tapers: 5,9 Window: 3s, 1s shift	ABS (A)
S2A, B	Same as Figure 2					
S2C	C57BL6 (7 recordings)	4M	Neuronexus A1x32Edge	Virtual reality	Tapers: 5,9 Window: 3s, 1s shift	ABS (A)
S3A, B	Same as Figure 3A-B					
S3C, D	C57BL6 (6 recording)	2 M	Neuronexus A1x32 (sites 25μm apart)	Contrast reversing checkerboard (3A; 15^0 squares, 2 cycles/s) or gray screen (3B)	Tapers: 5,9 Window: 3s, 1s shift	RD (B)
S4	Same as Figure 3C-D					
S5	Same as Figure 3E-F					
S6	C57BL6 (1 recordings)	1M	Neuronexus A2x4 tet	Gray screen	Tapers: 5,9 Window: 2.3s, 1s shift	ABS (A)
S7A-C	GAT2-Cre (8 recordings)	8 (M/F)	Neuronexus Buzsaki16	Full-field moving grating	Tapers: 3, 5 or 10; Window: 1s, 0.05s shift	KR (C)
S7E-I	VGAT-ChR2	2 (M/F)	Neuronexus Buzsaki32	Full-field moving grating	N/A	TL (C)
S7J-L	Same as Figure 4					

Experimental procedures

Experimental methods

The experiments were performed in three different laboratories, following one of three protocols below. We list the protocol followed for each experiment in Supplementary Table 1.

Protocol A: All procedures were conducted in accordance with the UK Animals Scientific Procedures Act (1986). Experiments were performed at University College London under personal and project licenses released by the Home Office following appropriate ethics review. We used both male and female mice of two strains: C57BL6 (17 animals) and PV-Ai32 (2 animals; RRID:IMSR_JAX:008069 / RRID:IMSR_JAX:012569) (full listing in Supplementary Table 1). Experimental methods are described in detail in Ayaz et al. 2013, Haider et al. 2013 and Saleem et al. 2013(Ayaz et al.,2013; Haider et al.,2013; Saleem et al.,2013). Briefly, animals were chronically implanted with a custom-built head post and recording chamber (3-4 mm inner diameter) under isoflurane anesthesia. They were then allowed to recover for 3 days with oral analgesic Rimadyl. Following recovery, the animals were acclimatized for 3-4 days to the head fixation apparatus. A craniotomy was performed between 4 – 24 hrs prior to the first recording session. Whole-cell patch-clamp recordings of EPSCs and IPSCs were performed with a cesium-based internal solution, with QX-314 (0.5mM) and tetraethylammonium (TEA) (5mM) included to block voltage-gated Na+ and K+ conductances. Extracellular spikes were isolated using the KlustaView Suite(Rossant et al.,2016). Visual stimuli were presented on LCD monitors that were gamma corrected. For most experiments, the mean light intensity level presented on the monitors was ~50 cd/m^2. The experiments in the dark were conducted making sure that all non-essential equipment was turned off, and essential equipment producing any light were covered with a filter (787 Marius Red; LEE filters) that only allows light that is not visible to mice. The light intensity level under these conditions was <10^{-2} cd/m^2, below the sensitivity of the light meter. For experiments in Figure 5 and Supplementary Figure S6, we placed Fresnel lenses in front of the three monitors to ensure that the light intensity entering the eye is independent of the viewing angle on the LCD monitors. With Fresnel lenses the light intensity level could vary from ~0.2 cd/m^2 to ~95 cd/m^2.

Protocol B: All procedures were performed on awake, adult mice and complied with the European Communities Council Directive 2010/63/EC and the German Law for Protection of Animals. All procedures were approved by the local authorities following appropriate ethics review. We used both male and female C57BL6 mice (full listing in Supplementary Table 1). Experimental methods are described in detail in Erisken et al, 2015 (Erisken et al.,2014). Briefly, animals were chronically implanted with a custom-built head post and recording chamber (3-4 mm inner diameter) under isoflurane anesthesia. Animals were then allowed to recover for a minimum of 3 days during which they received analgesics (Carprofen, 5 mg/kg, s.c.) and antibiotics (Baytril, 5 mg/kg, s.c.). Following complete recovery, the animals were handled and acclimatized for a minimum of ~1 week to head fixation and the spherical treadmill. One day before the first recording session, a craniotomy was performed under isoflurane anesthesia. Spikes were isolated using the Klusters suite (Hazan et al.,2006) in combination with KlustaKwik (Schmitzer-Torbert et al.,2005). Visual stimuli were presented on LCD monitors that were gamma corrected. For most experiments, the mean light intensity level presented on the monitors was ~50 Cd/m^2.

Protocol C: All procedures were conducted in accordance with the National Institutes of Health guidelines and with the approval of the Committee on Animal Care at UCSD (protocol S02160M). Animals were housed on a reverse light cycle in cages of four mice or less. At the time of electrophysiology, all animals were older than 3.5 weeks. Both male and female animals were used in an approximately equal ratio.

Experimental methods are described in detail in Lien et al. 2013 and Reinhold et al. 2015 (Lien and Scanziani,2013; Reinhold et al.,2015). Briefly, simultaneous V1 L4 intracellular whole-cell and LGN extracellular (Buzsaki32 probe, Neuronexus) recordings were performed in VGAT-ChR2 mice (JAX 014548 Zhao, et al; RRID:IMSR_JAX:014548) under urethane (1.5g/kg, IP)/chlorprothixene (2-4mg/kg, IP) anesthesia. V1 neurons were held in voltage clamp at -70 mV to record excitatory currents. Full field drifting bar gratings (100% contrast, 2 Hz temporal frequency, 0.04 cycles/deg spatial frequency) were presented on a monitor in the right visual field for 2.3 s preceded by 0.7 s of a gray screen of mean light intensity . Optogenetic suppression of V1 was achieved by constant illumination of V1 with blue light from a 1mm fiber-coupled LED positioned several millimeters over the crantiotomy (455 nm, 20mW at the fiber tip, Doric) on interleaved trials starting 645 ms prior to grating onset and extinguishing after grating offset. For experiments inactivating the LGN, V1 extracellular (Buzsaki16 probe, Neuronexus) recordings were performed in awake Gad2-Cre mice (Jackson Labs stock number: 010802 (Taniguchi et al.,2011); RRID:IMSR_JAX:010802) expressing Cre-dependent ChR2 (AAV2/1.CAGGS.flex.ChR2.tdTomato.SV40, Addgene 18917 (Boyden et al.,2005), from the University of Pennsylvania viral vector core) stereotactically confined to the thalamic reticular nucleus (TRN). Mice were head-fixed but free to spontaneously run or rest on a circular treadmill. Full field drifting bar gratings (100% contrast, 2 Hz temporal frequency, 0.04 cycles/deg spatial frequency) were presented on a monitor in the right visual field for 3 s preceded by 2.5 s of a gray screen of mean light intensity . Optogenetic suppression of LGN was achieved by constant illumination of TRN with blue light from a 473 nm laser coupled to an optical fiber of diameter 200 microns, the tip of which was positioned just above the TRN (stereotactic coordinates of tip: [1,540 µm posterior, 2,235 µm lateral, 3,158 µm ventral to bregma], 10mW at the fiber tip). Laser illumination of the TRN on interleaved trials starting 0.2 ms after grating onset and lasting 1 second led to a 70% suppression (Reinhold et al.,2015) of spiking in LGN over the duration of illumination. Single units from both LGN and V1 recordings were isolated using UltraMegaSort (Hill et al.,2011). Visual stimuli were presented on an LCD monitor that was gamma corrected. The mean light intensity level presented on the monitors was 75 Cd/m^2 for experiments in Figure 4.

Analyses

Spectral Analysis

We used the Chronux toolbox (http://chronux.org/), which is based on multi-taper methods, for spectral analyses. We measured the spectrogram of the local field potential using the function *mtspecgramc*. The power spectrum was calculated as the mean spectrogram across time. For spiking spectra, we used *mtspecgrampt* for the spike times (measured at 1 msec temporal resolution). We list the parameters we used for the spectral analysis (number of tapers and temporal window) in Supplementary Table 1. To calculate coherence across cells pairs, we used the Chronux function *mtcohgrampt*. To measure phase differences for any cell, we calculated its coherence with the pooled spike times from all other units. We calculated phase as the angle of the mean joint power across time. For Figure 5 and Supplementary Figure S6, we pooled the spike times across all units

detected on a tetrode to calculate the spiking spectrogram. There were a total of 12 recording sessions in three animals where we recorded responses to varying levels of light intensity, two of these sessions had more than one tetrode in the LGN. There were a total of 8 recording sessions where we recorded responses to varying levels of contrast, one of which had more than one tetrode in the LGN.

Residual power and peak frequency

We characterised the narrowband frequency using the residual power spectrum: the fractional increase in the power of the spectrum compared to a smoothed prediction (Supplementary Figure S1). We first calculated the smoothed prediction by fitting a 4th order polynomial to the spectrum in the range 20-55 Hz and 70-90 Hz. This prediction generally captured the 1/f fall-off and the broadband gamma peak. We next calculated the residual spectrum at each frequency as:

$$Residual\ spectrum = \frac{Spectrum - Prediction}{Prediction}$$

We define the narrowband frequency of any recording as the frequency with the maximum power of the residual spectrum in the range 55-70Hz. For spiking spectra from the LGN, we refer to the peak value of residual spectrum as the 'residual gamma power'.

To statistically compare peak narrowband gamma frequencies between different LED flicker rates (Fig. 1D), we divided the recordings for each flicker rate into 5 equal subsets, computed a peak frequency in each subset, and compared these using paired t-tests for each pair of flicker rates.

Comodulation

To access how the power of different frequency components of the local field potentials varied together, we calculated the comodulation: the correlation of the spectral power of the two frequencies across time. We measured this at all pairs of frequencies in the range of 20-90 Hz.

Cross-correlation

When computing cross-correlograms for neurons from the same recording tetrode (Fig. 3F), the central bin was discarded and interpolated to avoid spike collision artifacts.

Supplementary references

Ayaz, A., Saleem, A.B., Scholvinck, M.L., and Carandini, M. (2013). Locomotion controls spatial integration in mouse visual cortex. Curr Biol 23, 890-894.

Boyden, E.S., Zhang, F., Bamberg, E., Nagel, G., and Deisseroth, K. (2005). Millisecond-timescale, genetically targeted optical control of neural activity. Nat Neurosci 8, 1263-1268.

Erisken, S., Vaiceliunaite, A., Jurjut, O., Fiorini, M., Katzner, S., and Busse, L. (2014). Effects of locomotion extend throughout the mouse early visual system. Curr Biol 24, 2899-2907.

Haider, B., Hausser, M., and Carandini, M. (2013). Inhibition dominates sensory responses in the awake cortex. Nature 493, 97-100.

Hazan, L., Zugaro, M., and Buzsaki, G. (2006). Klusters, NeuroScope, NDManager: a free software suite for neurophysiological data processing and visualization. Journal of neuroscience methods 155, 207-216.

Hill, D.N., Mehta, S.B., and Kleinfeld, D. (2011). Quality metrics to accompany spike sorting of extracellular signals. J Neurosci 31, 8699-8705.

Lien, A.D., and Scanziani, M. (2013). Tuned thalamic excitation is amplified by visual cortical circuits. Nat Neurosci 16, 1315-1323.
Reinhold, K., Lien, A.D., and Scanziani, M. (2015). Distinct recurrent versus afferent dynamics in cortical visual processing. Nat Neurosci 18, 1789-1797.
Rossant, C., Kadir, S.N., Goodman, D.F., Schulman, J., Hunter, M.L., Saleem, A.B., Grosmark, A., Belluscio, M., Denfield, G.H., Ecker, A.S.*, et al.* (2016). Spike sorting for large, dense electrode arrays. Nat Neurosci 19, 634-641.
Saleem, A.B., Ayaz, A., Jeffery, K.J., Harris, K.D., and Carandini, M. (2013). Integration of visual motion and locomotion in mouse visual cortex. Nat Neurosci 16, 1864-1869.
Schmitzer-Torbert, N., Jackson, J., Henze, D., Harris, K., and Redish, A.D. (2005). Quantitative measures of cluster quality for use in extracellular recordings. Neuroscience 131, 1-11.
Taniguchi, H., He, M., Wu, P., Kim, S., Paik, R., Sugino, K., Kvitsiani, D., Fu, Y., Lu, J., Lin, Y.*, et al.* (2011). A resource of Cre driver lines for genetic targeting of GABAergic neurons in cerebral cortex. Neuron 71, 995-1013.
